# An heregulin-EGFR-HER3 autocrine signaling axis can mediate acquired lapatinib resistance in HER2+ breast cancer models

**DOI:** 10.1186/bcr3480

**Published:** 2013-09-18

**Authors:** Wenle Xia, Emanual F Petricoin, Sumin Zhao, Leihua Liu, Takuya Osada, Qing Cheng, Julia D Wulfkuhle, William R Gwin, Xiaoyi Yang, Rosa I Gallagher, Sarah Bacus, H Kim Lyerly, Neil L Spector

**Affiliations:** 1 Department of Medicine, Medical Science Research Building 1, Research Drive, Duke University Medical Center, Durham, NC 27710, USA; 2 Duke Cancer Institute, Duke University Medical Center, Durham, NC 27710, USA; 3 Center for Applied Proteomics and Molecular Medicine, George Mason University, 10900 University Blvd, Manassas, VA 20155, USA; 4 Department of Surgery, Medical Science Research Building 1, Research Drive, Duke University Medical Center, Durham, NC 27710, USA; 5 Targeted Molecular Diagnostics/Quintiles, 610 Oakmont Lane, Westmont, IL 60559, USA

## Abstract

**Introduction:**

The human epidermal growth factor receptor 2 (*HER2*) receptor tyrosine kinase (RTK) oncogene is an attractive therapeutic target for the treatment of HER2-addicted tumors. Although lapatinib, an FDA-approved small-molecule HER2 and epidermal growth factor receptor (EGFR) tyrosine kinase inhibitor (TKI), represents a significant therapeutic advancement in the treatment of HER2^+^ breast cancers, responses to lapatinib have not been durable. Consequently, elucidation of mechanisms of acquired therapeutic resistance to HER-directed therapies is of critical importance.

**Methods:**

Using a functional protein-pathway activation mapping strategy, along with targeted genomic knockdowns applied to a series of isogenic-matched pairs of lapatinib-sensitive and resistant cell lines, we now report an unexpected mechanism of acquired resistance to lapatinib and similar TKIs.

**Results:**

The signaling analysis revealed that whereas HER2 was appropriately inhibited in lapatinib-resistant cells, EGFR tyrosine phosphorylation was incompletely inhibited. Using a targeted molecular knockdown approach to interrogate the causal molecular underpinnings of EGFR-persistent activation, we found that lapatinib-resistant cells were no longer oncogene addicted to HER2-HER3-PI3K signaling, as seen in the parental lapatinib-sensitive cell lines, but instead were dependent on a heregulin (HRG)-driven HER3-EGFR-PI3K-PDK1 signaling axis. Two FDA-approved EGFR TKIs could not overcome HRG-HER3-mediated activation of EGFR, or reverse lapatinib resistance. The ability to overcome EGFR-mediated acquired therapeutic resistance to lapatinib was demonstrated through molecular knockdown of EGFR and treatment with the irreversible pan-HER TKI neratinib, which blocked HRG-dependent phosphorylation of HER3 and EGFR, resulting in apoptosis of resistant cells. In addition, whereas HRG reversed lapatinib-mediated antitumor effects in parental HER2^+^ breast cancer cells, neratinib was comparatively resistant to the effects of HRG in parental cells. Finally, we showed that HRG expression is an independent negative predictor of clinical outcome in HER2^+^ breast cancers, providing potential clinical relevance to our findings.

**Conclusions:**

Molecular analysis of acquired therapeutic resistance to lapatinib identified a new resistance mechanism based on incomplete and "leaky" inhibition of EGFR by lapatinib. The selective pressure applied by incomplete inhibition of the EGFR drug target resulted in selection of ligand-driven feedback that sustained EGFR activation in the face of constant exposure to the drug. Inadequate target inhibition driven by a ligand-mediated autocrine feedback loop may represent a broader mechanism of therapeutic resistance to HER TKIs and suggests adopting a different strategy for selecting more effective TKIs to advance into the clinic.

## Introduction

Members of the human epidermal growth factor receptor (HER) family of transmembrane receptor tyrosine kinases (HER1/EGFR; HER2; HER3; HER4) and their respective ligands constitute a robust biologic system that plays a key role in the regulation of cell-proliferative growth, survival, and differentiation [1-6]. Ligand-bound monomeric HER receptors form homo- or heterodimers, which in turn activate their respective autokinase activities, leading to self-phosphorylation of c-terminus tyrosine residues serving as docking sites for adaptor proteins that activate downstream growth and survival signaling cascades [3-6]. HER2, the preferred dimerization partner for HER3 and EGFR, amplifies the signal generated through the dimer receptor complex [4]. HER3, conversely, is transactivated by its dimerization partner [7]. Importantly, HER3 contains six phosphotyrosine binding sites for the p85 subunit of PI3K (phosphoinositide 3-kinase), the most of all HER family members [8]. Consequently, HER2-HER3 dimers are potent activators of PI3K signaling, which in breast and other solid tumors, represents an important oncogenic signaling unit [9].

Deregulation of HER signaling, which can occur as a consequence of gene amplification (HER2) or gain-of-function mutation (EGFR) promotes solid-tumor oncogenesis. In breast and ovarian cancers, HER2 overexpression (HER2+) predicts for a poor clinical outcome [10,11], findings that have prompted the development of HER2-targeted therapies, including small-molecule tyrosine kinase inhibitors (TKIs) designed to block the autokinase activity of the HER2 receptor. Lapatinib is a highly selective, small-molecule inhibitor of the HER2 and EGFR tyrosine kinases [12]. It is currently the only FDA-approved tyrosine kinase inhibitor (TKI) for the treatment of advanced-stage HER2^+^ breast cancers [13]. Although lapatinib is considered an equipotent inhibitor of HER2 and EGFR, based on data from *in vitro* kinase assays [14,15], its clinical efficacy to date has been limited to HER2^+^ breast cancers [16]. Despite representing a significant therapeutic advance in the treatment of aggressive HER2^+^ breast cancers, the clinical efficacy of lapatinib has been limited by the inevitable development of therapeutic resistance [13,16]. In this regard, several mechanisms of acquired therapeutic resistance have been reported, based primarily on data generated from preclinical models [17-23]. In contrast to other kinase inhibitors, in which mutations in the ATP-binding pocket of the targeted kinase can lead to reactivation of the targeted protein [24,25], we and others have shown that HER2 mutation do not appear to play a role in resistance, and that phosphorylation of HER2 remains inhibited in models of acquired lapatinib resistance [17,22,23]. Furthermore, previous work from our laboratory has shown that molecular knockdown of HER2 does not reverse lapatinib resistance, providing additional evidence that resistant cells are no longer dependent on HER2 for survival [17]. The recent decision to discontinue a lapatinib monotherapy treatment arm in the ALTTO study, an ongoing global phase III clinical trial of adjuvant HER2-targeted therapies in the treatment of early-stage HER2^+^ breast cancers, due to an increased incidence of disease recurrence, underscores the need to understand better the resistance conundrum. Elucidating mechanisms of acquired therapeutic resistance to HER TKIs and kinase inhibitors in general is therefore of critical importance in the management of kinase-driven diseases.

The tumor-promoting PI3K cell-signaling pathway has been shown to be persistently activated in models of acquired therapeutic resistance to lapatinib and similar HER TKIs in class [19,20]. The role of activating PI3KCA mutations or PTEN loss as a potential explanation for the persistent activation of PI3K signaling in lapatinib resistance remains controversial [19,26-28]. Here, we show that acquired therapeutic resistance to lapatinib in models of HER2^+^ breast cancer can be mediated by autocrine induction of the membrane-bound form of the HER3 ligand heregulin (HRG). Increased expression of full-length HRG in combination with inadequate inhibition of EGFR phosphorylation by lapatinib promotes an HRG-HER3-EGFR-PI3K signaling axis that contributes not only to lapatinib resistance, but also to cross-resistance to FDA-approved EGFR TKIs. These findings could have a significant impact not only on the treatment of HER2- and EGFR-dependent tumors, but also on relevance to the treatment of kinase-driven diseases in general.

## Methods

### Cell culture and reagents

Human breast cancer cell lines BT474, SKBR3, Au565, and SUM190 were obtained from the American Type Culture Collection (Manassas, VA, USA). Lapatinib-resistant cell lines (rBT474, rSKBR3, rAu565, and rSUM190) were generated and continuously maintained in 1 μ*M* lapatinib, as previously described [17,18]. The 4G10 anti-phosphotyrosine (p-tyr) antibody was purchased from Sigma-Aldrich (St. Louis, MO, USA). Monoclonal antibodies to c-HER2 and EGFR were purchased from Neo Markers (Union City, CA, USA). Phospho-specific primary antibodies to EGFR (Y992), EGFR (Y1148), EGFR (Y1173), EGFR (Y1068), and HER3 (Y1197), and PARP cleavage product were obtained from Cell Signaling (Beverly, MA, USA). Anti-PDK1 antibody was purchased from R&D Systems (Minneapolis, MN, USA). Antibodies to phosho-PI3Kp85 (Y508), Akt1/2, phospho-Akt1/2 (S473), phospho-Akt1/2 (T308), HRG and siRNA constructs (Akt1/2; PI3K subunits; PKC, PDK1, SGK, HRG, and control siRNA-A) were purchased from Santa Cruz (Santa Cruz, CA, USA). The PHLPP2 antibody was from Bethyl (Montgomery, TX, USA). ADAM17 antibody was purchased from Abcam (Cambridge, MA, USA). Erlotinib was obtained from Genentech (South San Francisco, CA, USA). SU11274, neratinib, and AZD0530 were from Selleck (Houston, TX, USA). IRDye800 conjugated affinity-purified anti-rabbit IgG and anti-mouse IgG were purchased from Rockland (Gilbertsville, PA, USA). Alexa Fluor680 goat anti-rabbit IgG was obtained from Molecular Probes (Eugene, OR, USA). NVP-BEZ 235 was obtained from Novartis (Basel, Switzerland). Lapatinib and gefitinib were purchased from LC Laboratories (Woburn, CA, USA).

### siRNA transfection

Cell transfections were performed in a six-well format by using 5 μl lipofectamine 2000 (Invitrogen Life Technologies) in OPTI-MEM I (Invitrogen Life Technologies, Carlsbad, CA, USA) at 5 × 10^5^ cells per well, with individual siRNAs against target proteins, and nonspecific siRNA (NSC) as controls, as described in the Invitrogen transfection protocol and in our previous publications [17,26]. The concentration of siRNA was 100 n*M* in a final volume of 2.5 ml. After 16 to 18 hours, the transfection media was removed and replaced with complete RPMI 1640 supplemented with 1 μ*M* lapatinib for an additional 48 hours.

### SDS-PAGE and Western blot analysis

Details of the methods used for SDS-PAGE and Western blot analysis have been previously described [17,18,26]. In brief, membranes were incubated with primary antibodies, washed several times in PBS, and then incubated with a fluorescence-conjugated secondary antibody at a 1:10,000 dilution with 5% try milk in PBS for 60 minutes, protected from light. After washing in PBS + 0.1% tween-20, the membranes were scanned and visualized by using the Odyssey Infrared Imaging System (LI-COR, Inc., Lincoln, NE, USA).

### Cell growth and viability assay

The cell-growth assay was performed in a 96-well plate format in a final volume of 100 μl/well cell-culture medium with the cell-proliferation reagent WST-1 from Roche Diagnostics (Mannheim, Germany). Details of the WST-1 assay were previously described [17,18,26].

### Reverse-phase protein microarray construction and analysis

Reverse-phase protein microarrays were constructed as described previously [29]. A list of the antibodies used in this analysis and their sources are provided (see Additional file 1). In brief, denatured lysates were spotted onto nitrocellulose-coated glass slides (Whatman, Inc, Sanford, ME, USA) by using a 2470 Arrayer (Aushon BioSystems, Burlington, MA, USA), outfitted with 185-μm pins. Each sample was printed in triplicate as a neat and 1:4 dilution two-point dilution series to ensure that one of the points was in the linear dynamic range of the fluorescence assay. A high and low internal control for antibody-staining specificity, consisting of lysates derived from pervanadate-treated HeLa cells and calyculin-treated Jurkat cells were used and spotted onto every array, along with the experimental samples. Slides were stored desiccated at -20°C until staining. Blocked arrays were stained with antibodies on an automated slide stainer (Dako, Carpinteria, CA, USA) by using the Catalyzed Signal Amplification System kit (CSA; Dako) and streptavidin-conjugated IRDye680 (LI-COR Bioscience, Lincoln, NE, USA) to generate a fluorescence signal. Each antibody used in the staining process was previously validated by using Western blot procedure. Antibodies producing a single band in correspondence to the molecular weight of interest were considerate validated and eligible for use in immunostaining. All intensity values were normalized to total protein for each sample, to account for differences in intensity due solely to starting lysate concentration variance. The total amount of protein present in each sample was estimated through Sypro Ruby Protein Blot Stain (Molecular Probes) according to the manufacturer’s instructions, as previously described [29]. All Sypro and immunostained slides were scanned by using a Revolution 4550 scanner (Vidar Corp., Herndon, VA, USA), and acquired images were analyzed by using MicroVigene v2.9.9.9 (VigeneTech, Carlisle, MA, USA) that performed spot finding, local background subtraction, replicate averaging, and total protein normalization, producing a single value for each sample at each end point. Statistical analysis of the array data was performed by *T* testing or Wilcoxon two-sample rank-sum test by using R v2.9.2 (R Foundation for Statistical Computing, Vienna, Austria) to compare values between groups, depending on normalcy distribution values. *P* values < 0.05 were considered statistically significant.

### Immunofluorescence microscopy

Cells were cultured on cover glass in six-well plates. After washing with PBS, cells were fixed and permeabilized with methanol/acetone (1:1) and blocked with 2% goat serum, 0.3% triton X-100 in PBS at room temperature, followed by washing with PBS, and incubated with an anti-HRG antibody at 4°C. After extensive washings, the cells were incubated with anti-rabbit IgG conjugated with Alexa Fluor 555 (Cell Signaling, Danvers, MA, USA) followed by a liquid mountant application with ProLong Gold anti-fade reagent with DAPI nuclear stain (Life Technologies, Grand Island, NY, USA). A Zeiss Axio Observer was used for photographs.

### Gene-expression data analysis

We compiled a collection of 4,010 breast tumor gene-expression data derived from 23 datasets that have been posted on the NCBI Gene Expression Omnibus (GEO) database, as previously described [30]. In addition to the raw expression data, we also obtained recurrence-free survival data from a subset of the samples (*n* = 1,372).

HRG (NRG1) expression was measured by probe set 208231_at. We assigned each of 4,010 sample into Low (first quartile, lowest 25%), Intermediate (second quartile, intermediate 50%), and High (third quartile, highest 25%) subgroups, according to HRG expression levels, and compared prognosis differences among these subgroups by using Kaplan-Meier estimates of recurrence-free survival analysis. Furthermore, we applied HRG expression signal as continuous variable and determined correlation of HRG expression and risk of recurrence among 204 HER2^+^ breast cancer samples, by using Cox-regression survival analysis.

### Statistical analysis

Data were expressed as means with standard error bars included. The Student *t* test was used to determine statistical significance between two groups. A value of *P* < 0.05 was considered a statistically significant difference.

## Results

### PI3K-pathway signaling is persistently activated in lapatinib-resistant breast cancer cells

**Figure 1:**
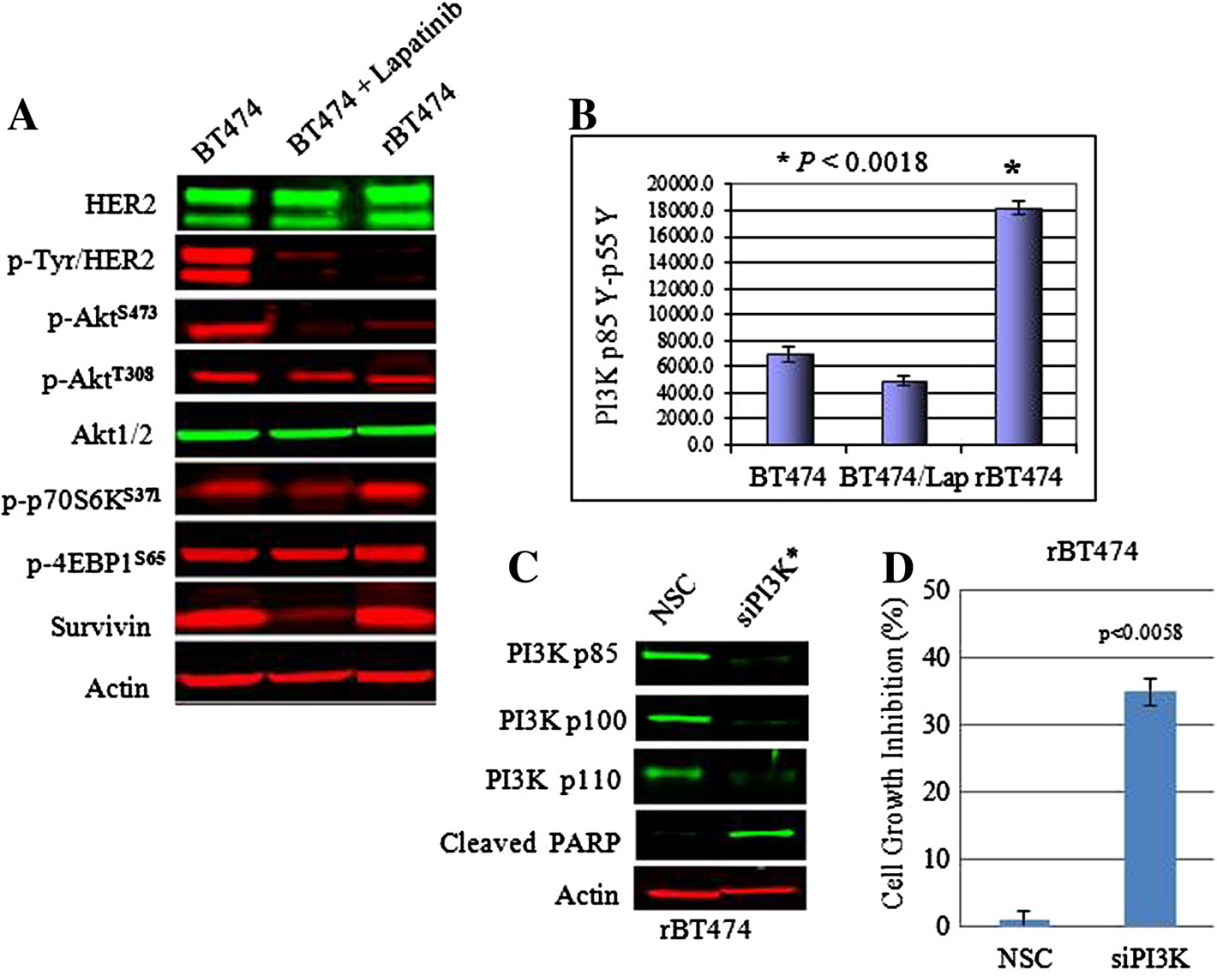
**Persistent activation of PI3K signaling promotes survival in lapatinib-resistant cells. (A)** pHER2, total HER2, Akt^**T308**^, Akt^**S473**^, p70S6K^**S371**^, 4EBP1^**S65**^, and survivin steady-state protein expression in untreated parental BT474, BT474 treated with 0.5 μ*M* lapatinib for 48 hours, and rBT474 maintained in 1 μ*M* lapatinib, as determined by Western blot analysis from whole cell extracts. **(B)** Phospho-PI3K protein expression was determined by RPMA in the same treatment groups as described in **(A)**. Results represent the mean ± standard error of triplicate samples, and are representative of three independent experiments. *P < 0.0018. **(C)** Molecular knockdown of PI3K by using pooled siRNA against PI3K subunits (*) in rBT474 cells was confirmed by Western blot analysis by using subunit-specific antibodies and an anti-PARP cleavage-product antibody. Cells transfected with scrambled siRNA construct (NSC) served as controls. Actin steady-state protein levels served as a control to ensure equal loading of protein. The results are representative of three independent experiments. **(D)** The effects of siRNA-mediated knockdown of PI3K on rBT474 cell growth (*P <* 0.0058). Nonspecific siRNA construct (NSC) served as a control. Results represent the mean ± standard error of triplicate samples, and are representative of three independent experiments.

We used HER2^+^ breast cancer models of acquired therapeutic resistance to lapatinib established in our laboratory, as previously described [17,18] to investigate how, and to what extent, deregulation of the protein signaling network contributes to therapeutic resistance to HER2/EGFR TKIs. As previously shown, these cells are maintained in 1 μ*M* lapatinib without decreased viability, compared with parental cell counterparts that are sensitive to the antitumor effects of lapatinib (see Additional file 2). To determine the activation state of the cell-signaling network in lapatinib-resistant tumor cells, we evaluated the expression of >150 protein/phosphoproteins representing mediators of key cell processes by using quantitative reverse-phase protein arrays (RPMAs) [29]. Findings from the RPMA analysis were confirmed by Western blot analysis. For the purposes of the following studies, resistant cell lines were maintained in the continuous presence of 1 μ*M* lapatinib, even when combined with other treatments. Consistent with our previous findings [17], HER2 phosphorylation remained inhibited in lapatinib-resistant cells (Figure 1A). With this strategy, we found that the PI3K pathway remained activated in our models of acquired lapatinib resistance, as indicated by the persistent phosphorylation of PI3K-p85^**Y458**^, Akt^**T308**^, mTOR^**S2481**^, p70S6K^**S371**^, Bad^**S136**^, and 4EBP1^**S65**^ (Figure 1A, B; see Additional file 3). In addition, protein expression of survivin, a member of the inhibitor of apoptosis family whose downregulation in lapatinib-treated HER2^+^ breast cancer cells we had previously shown to correlate with lapatinib antitumor activity in a PI3K-dependent manner [31], remained intact in lapatinib-resistant cells.

### A PI3K-PDK1-Akt^T308^ signaling axis maintains the survival of lapatinib-resistant tumor cells

**Figure 2:**
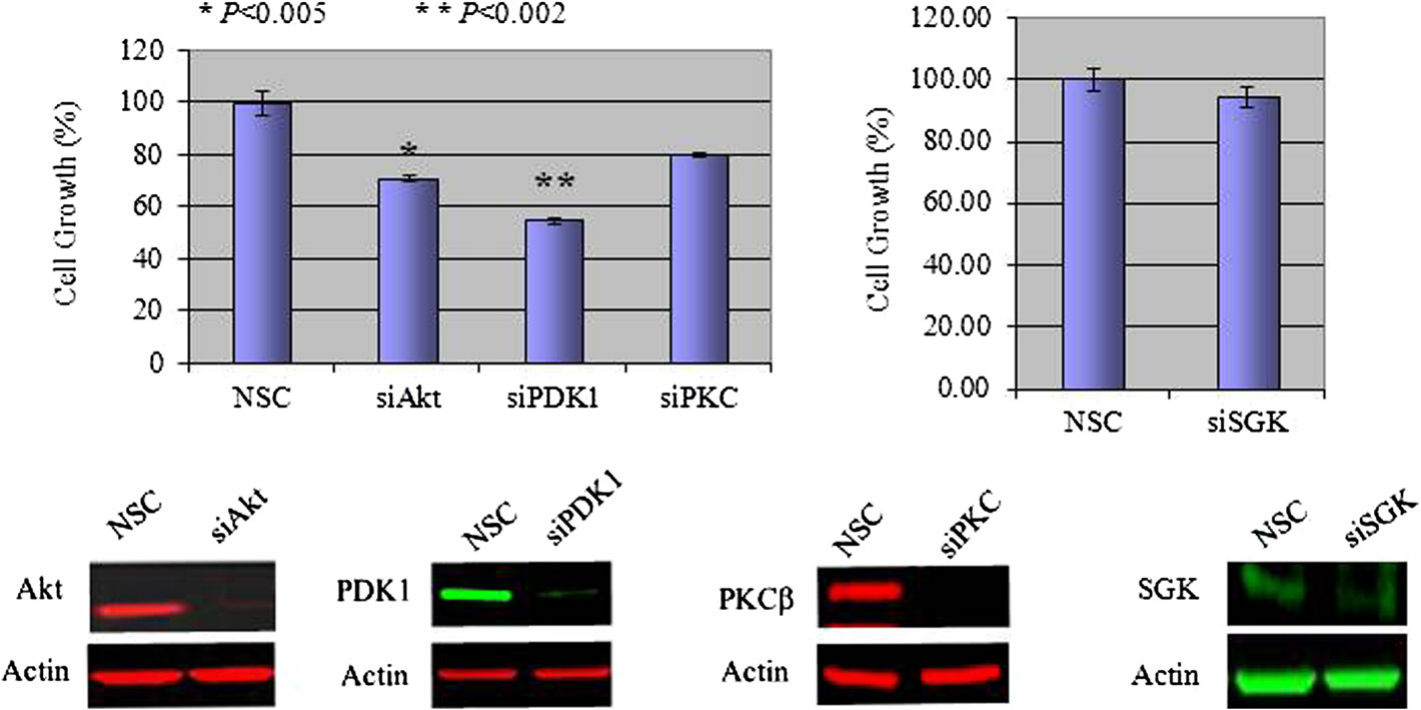
**A PI3K-PDK1 signaling axis mediates the effects of PI3K signaling in lapatinib-resistant cells.** A comparison of the effects of siRNA-mediated knockdown of Akt, PDK1, PKCβ, and SGK on the growth of rBT474 cells. Cells transfected with scrambled siRNA construct (NSC) served as controls. Growth assays were performed as described in Methods. Knockdown of targeted protein was confirmed by Western blot analysis (lower panels). Actin steady-state protein levels served as a control to ensure equal loading of protein. Results represent the mean ± standard error of triplicate samples and are representative of three independent experiments. *P < 0.005; **P < 0.002.

We used a molecular approach to knock down specific targeted proteins in the PI3K signaling pathway to determine the functional role of PI3K in maintaining the resistant phenotype. As shown, small interfering RNA (siRNA)-mediated knockdown of PI3K, primarily targeting the p110 catalytic subunit, and triggered resistant cells to undergo apoptosis, as indicated by increased expression of cleaved PARP and significant inhibition of cell growth and viability (*P* < 0.0058) (Figure 1C, D). A number of downstream intermediaries transduce the PI3K signaling effects (for example, Akt, PDK1, SGK, and PKCβ). Interestingly, phosphorylation of Akt serine 473 (S473), which is considered a hallmark of PI3K pathway activation, was inhibited in resistant cells despite persistent PI3K pathway activation (Figure 1A). Instead, phosphorylation of Akt threonine 308 remained intact, implying a role for PDK1, the kinase responsible for phosphorylating Akt^**T308**^ in resistant cells. To expand on these findings, we individually knocked down Akt, PDK1, SGK, and PKCβ to determine each of their effects on the viability of resistant cells. We found that knockdown of Akt or PDK1, but not PKCβ or SGK had a significant antitumor effect in lapatinib-resistant cells (Figure 2). The overlapping antitumor effects in response to knocking down Akt or PDK1 implicated the role of a PI3K-PDK1-Akt^**T308**^ signaling axis in maintaining the survival of lapatinib-resistant cells.

### The regulation of PI3K-pathway activation and cell survival is switched from HER2-HER3 in the treatment-naïve state to EGFR-HER3 signaling in lapatinib resistance

Lapatinib-naïve HER2+ breast cancer cells are addicted to HER2 signaling. Work from our laboratory and others has shown that regulation of prosurvival PI3K signaling in lapatinib-resistant breast cancer cells appears to be mediated through an HER2-independent mechanism(s) [17,32]. Although loss of the PTEN tumor suppressor, or the presence of PI3KCA gain-of-function mutations can lead to constitutive activation of PI3K signaling in breast cancer [33,34], neither was found to be relevant in our models of resistance (data not shown). Similar to that reported by others, we found that redundant survival pathways previously linked to HER TKI resistance were phosphorylated in our models of resistance (for example, c-src, c-met) [20,35,36]; however, we were unable to demonstrate their functional role in regulating the survival of resistant cells (see Additional files 4 and 5).

**Figure 3:**
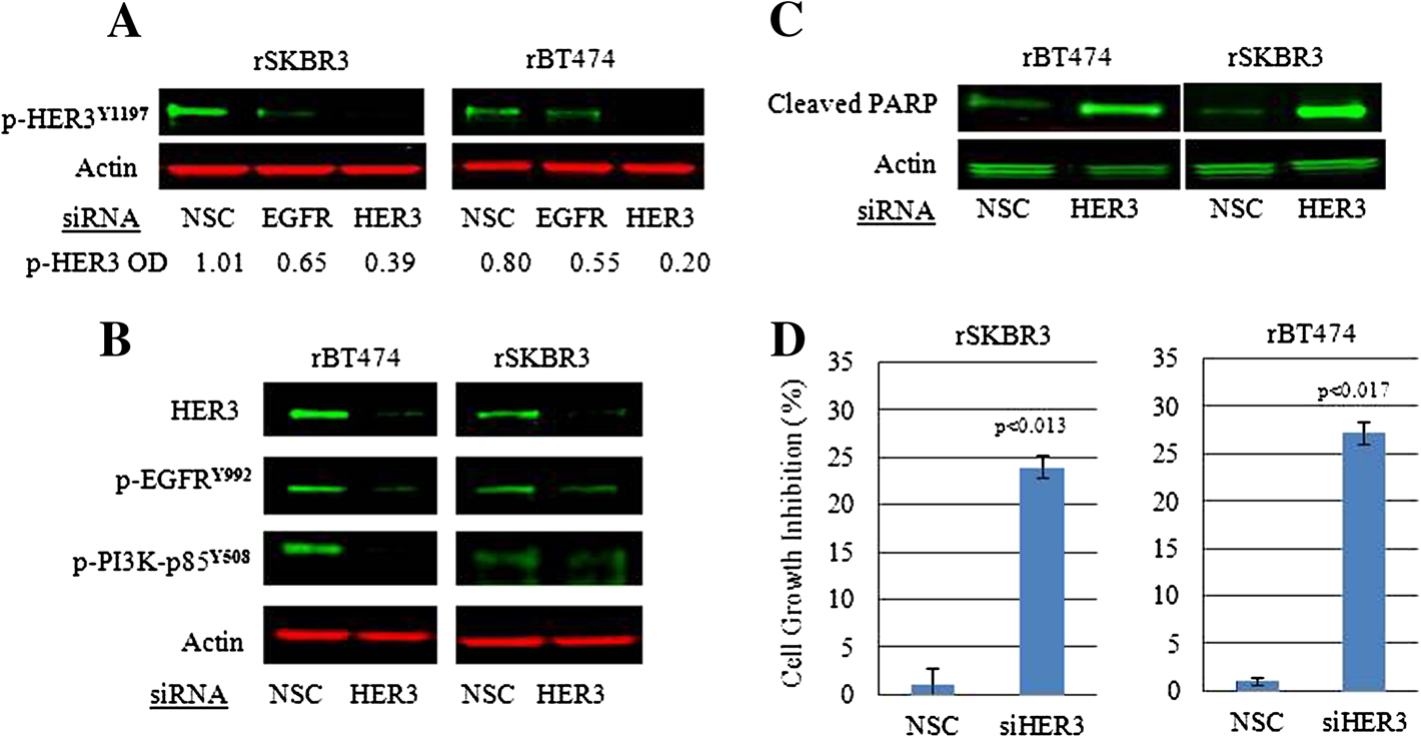
**EGFR-HER3 transactivation regulates resistant cell survival through PI3K signaling. (A)** The effects of EGFR and HER3 knockdown on steady-state phospho-HER3^**Y1197**^ protein expression. The absolute optical density (OD) values attributed to the p-HER3 bands, determined by using the Odyssey Infrared Imaging System, are indicated under each corresponding lane. **(B)** Western blot analysis with the indicated phosphospecific antibodies showing the effects of HER3 knockdown on EGFR^**Y992**^ and PI3K-p85^**Y508**^ in rBT474 and rSKBR3 cells. **(C)** Increased steady-state protein levels of cleaved PARP product, and **(D)** inhibition of cell growth in response to HER3 knockdown in rBT474 and rSKBR3 cells. *P* < 0.017 (rBT474); *P* < 0.013 (rSKBR3). Results represent the mean ± standard error of triplicate samples and are representative of three independent experiments.

**Figure 4:**
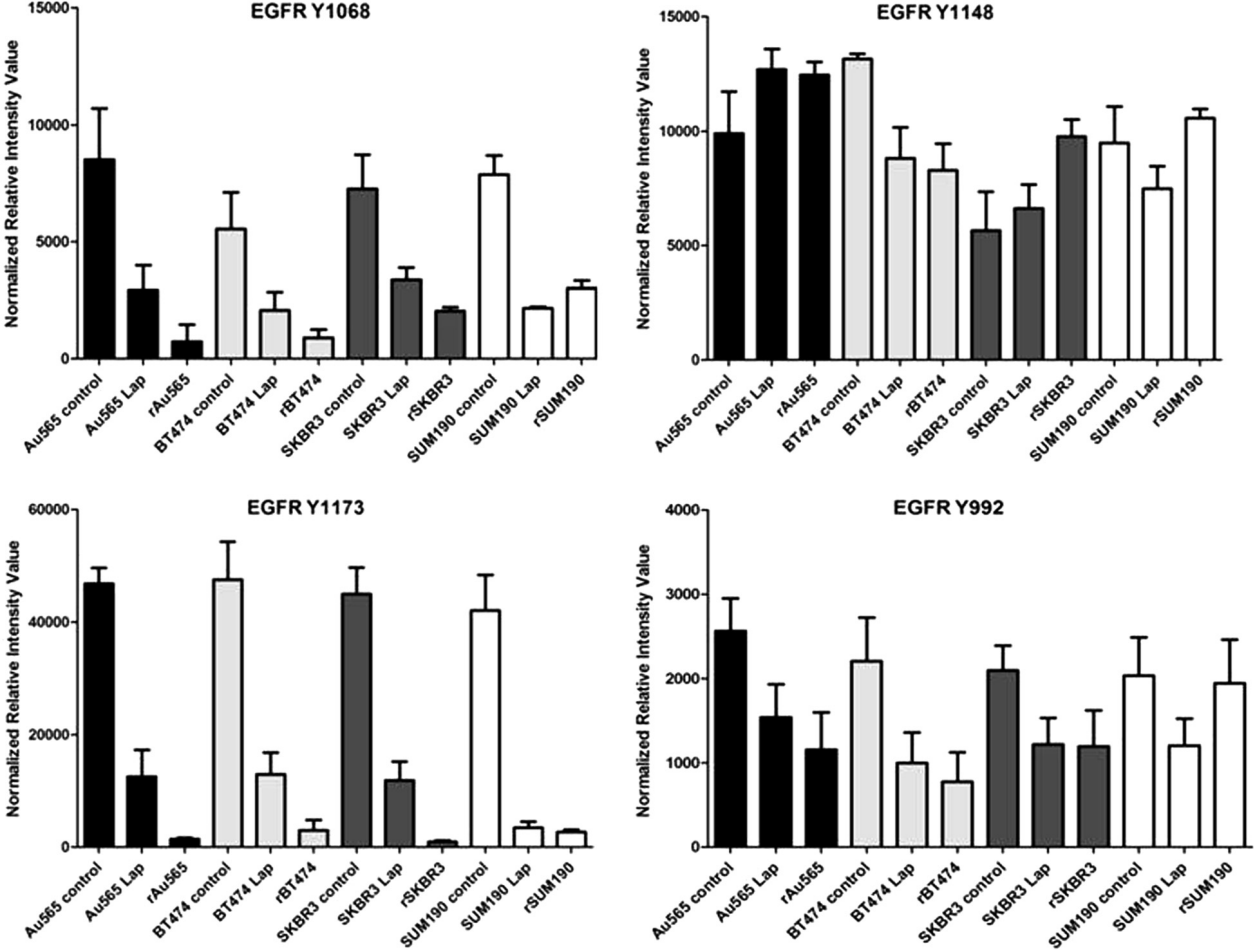
**Lapatinib-resistant cells exhibit a mixed pattern of EGFR tyrosine autophosphorylation.** Reverse-phase protein microarray analysis of EGFR Y992, Y1068, Y1148, and Y1173 in parental HER2+ breast cancer cell lines (BT474; SKBR3; Au565; SUM190), parental cells treated with 1 μ*M* lapatinib for 24 hours, and lapatinib-resistant cell counterparts (rBT474; rSKBR3; rAu565; rSUM190) maintained in 1 μ*M* lapatinib. Results represent the mean ± standard error of triplicate samples and are representative of three independent experiments.

HER2-HER3 heterodimers are potent activators of PI3K signaling [8]. HER3 was persistently phosphorylated on tyrosine 1197 in our models of lapatinib resistance (Figure 3A) despite inactivation of its preferred heterodimer partner HER2 (Figure 1A). HER3 knockdown in resistant cells led to inhibition of PI3K-p85^**Y508**^ phosphorylation, increased expression of cleaved PARP, and significant inhibition of cell growth and viability (*P* < 0.013 in rSKBR3; *P* < 0.017 in rBT474) (Figure 3A through D) revealing its central role in the maintenance of cell survival in our models. Unable to detect HER4 protein in resistant cells (data not shown), we speculated that EGFR, which is also expressed in lapatinib-resistant cells, might be responsible for the persistent transactivation of HER3 in resistant cells. Because lapatinib is reported to be an equipotent inhibitor of the HER2 and EGFR kinases [14,15], we expected to find that phosphorylation of EGFR, similar to HER2, would be inhibited in resistant cells. However, analysis of individual EGFR phosphotyrosine sites in lapatinib-resistant cells revealed a mixed pattern, as evidenced by variably persistent phosphorylation of tyrosines 992 and 1148, and marked inhibition of other phosphotyrosine sites (Y1173, Y1068) (Figure 4).

**Figure 5:**
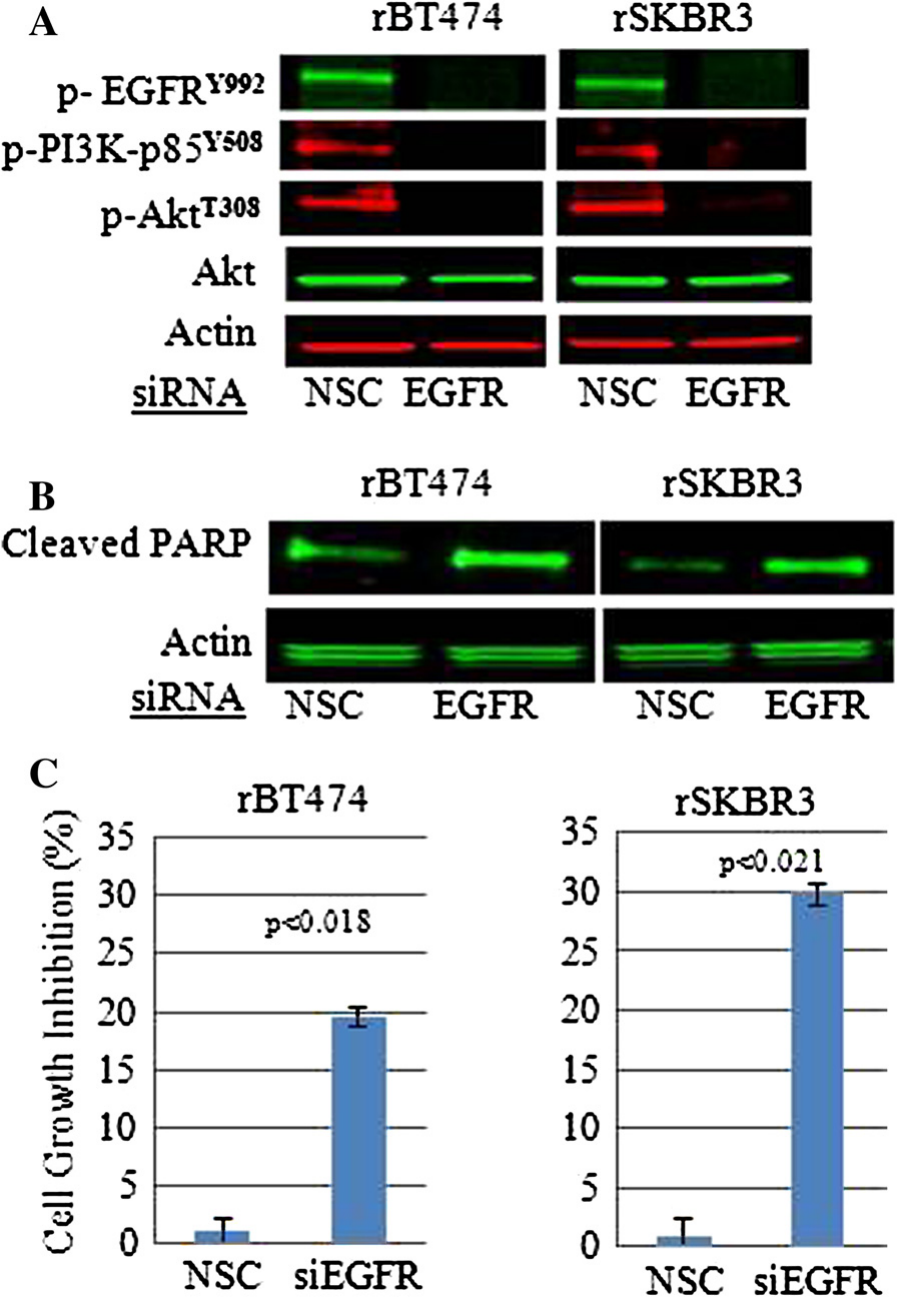
**The effects of EGFR knockdown on steady-state levels of the indicated proteins and phosphoproteins by using phosphospecific antibodies. (A)** Western blot analysis with the indicated phosphospecific antibodies showing the effects of EGFR knockdown on EGFR^**Y992**^, PI3K-p85^**Y508**^, and Akt^**T308**^ in rBT474 and rSKBR3 cells. **(B)** Western blot analysis of cleaved PARP product in rBT474 and rSKBR3 cells transfected with EGFR siRNA. Scrambled siRNA sequences (NSC) served as controls. Steady-state actin protein served as a control for equal loading of protein. Western blot data shown in A and B are representative of three independent experiments. **(C)** Effects of siRNA-mediated knockdown of EGFR on tumor cell growth in rBT474 and rSKBR3 cell lines. Results represent the mean ± standard error of triplicate samples and are representative of three independent experiments. *P* < 0.018 (rBT474) and *P* < 0.021 (rSKBR3).

These findings made it tempting to speculate that escape from, or incomplete inhibition of EGFR tyrosine autophosphorylation sites in response to lapatinib, over time, led to a switch in the regulation of cell survival from HER2-HER3-PI3K signaling in lapatinib-naive HER2^+^ breast cancer cells, to EGFR-HER3-PI3K in cells that become resistance to lapatinib. To test this hypothesis, we molecularly knocked down EGFR in lapatinib-resistant cells, which reduced HER3^**Y1197**^ phosphorylation and PI3K signaling, and led to increased apoptosis (cleaved PARP) with a statistically significant reduction in cell viability (*P* < 0.018 in rBT474; *P* < 0.021 in rSKBR3) (Figure 5A through C). Thus, the regulation of HER3 phosphorylation appears to switch from HER2 in treatment naïve cells, to EGFR in HER2^+^ breast cancer cell lines that have become resistant to lapatinib.

### Activation of a negative-feedback loop in resistant tumor cells specifically dephosphorylates Akt^S473^ despite persistent PI3K-pathway activation

Inhibition of Akt^**S473**^ phosphorylation in resistant cells appeared inconsistent with the persistent activation of the PI3K signaling pathway (Figure 1A). In this context, PHLPPL (PH domain leucine-rich repeat-containing phosphatase-like) is a protein phosphatase that is transcriptionally regulated by mTORC1 [37,38]. PHLPPL negatively feeds back on PI3K signaling by selectively dephosphorylating Akt on S473, not T308 [37,38], making it tempting to speculate that PHLPPL might be responsible for the pattern of Akt phosphorylation observed in lapatinib-resistant cells. We found that expression of PHLPPL protein was increased in resistant cells compared with their parental cell counterparts (see Additional file 6A). PHLPPL protein expression was decreased in parental cells treated with 1 μ*M* lapatinib for 24 hours (Additional file 6A), consistent with inhibition of PI3K-mTOR signaling in lapatinib-treated parental cells. If the increased expression of PHLPPL in resistant cells were related to persistent PI3K-mTOR pathway activation, then inhibition of PI3K signaling should block PHLPPL expression. Indeed, PHLPPL expression was inhibited in resistant cells growing in the presence of 1 μ*M* lapatinib, after treatment with the dual PI3K-mTOR kinase inhibitor BEZ-NVP 235 [39] (Additional file 6A). In addition, molecular knockdown of EGFR, which blocked PI3K signaling, also inhibited PHLPPL protein expression (Additional file 6B). These findings suggest that Akt^**S473**^ phosphorylation may not necessarily represent a reliable pharmacodynamic readout to assess the effects of targeted therapies on PI3K signaling.

### EGFR represents an attractive target in lapatinib-resistant HER2^+^ breast cancer cells

**Figure 6:**
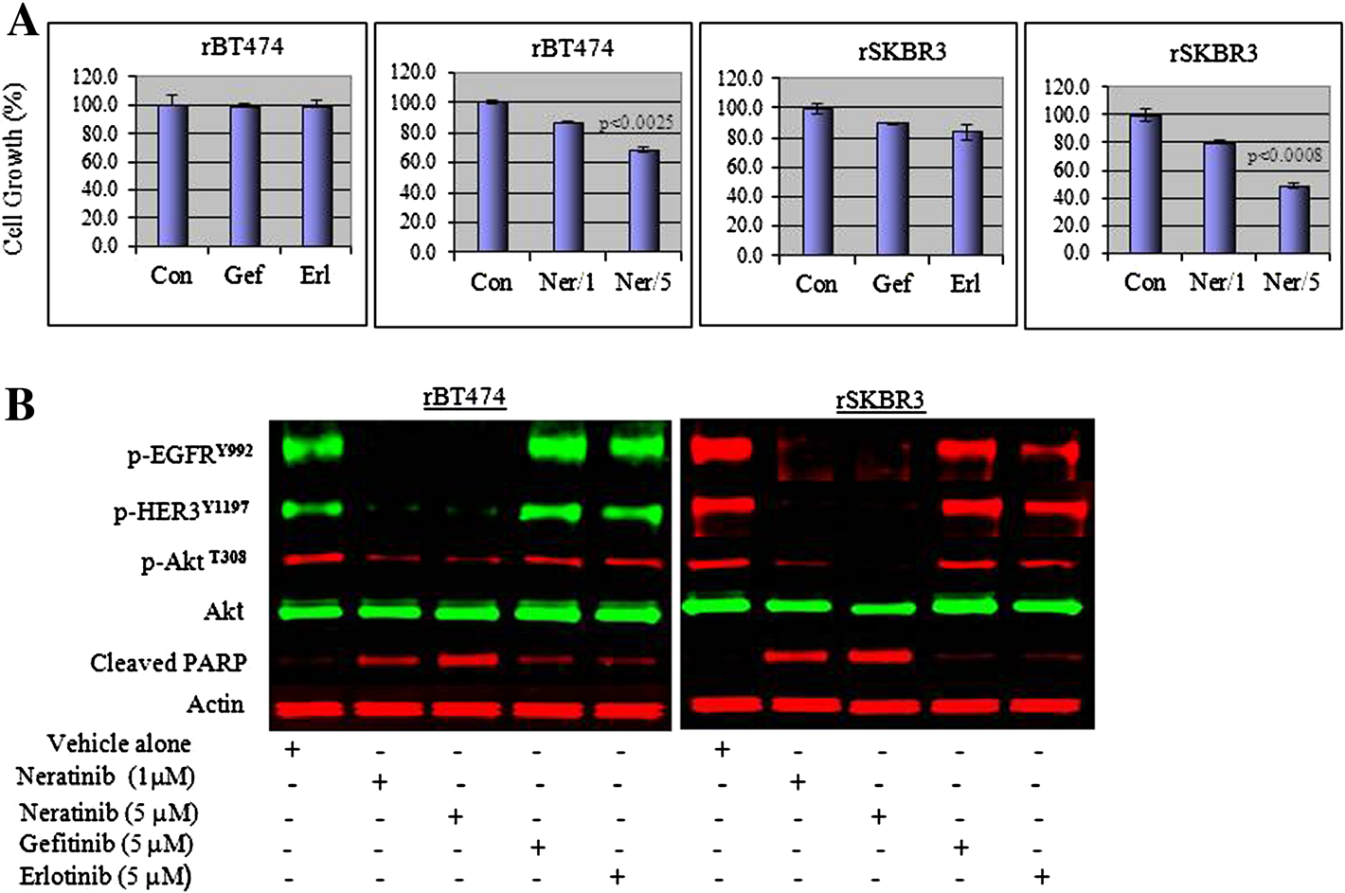
**Gefitinib and erlotinib do not reverse lapatinib resistance. (A)** Cell growth of rBT474 and rSKBR3 cells after treatment with 5 μ*M* gefitinib or erlotinib or 1 μ*M* or 5 μ*M* neratinib for 72 hours. Resistant cells were maintained in the presence of 1 μ*M* lapatinib. Results represent the mean ± standard error of triplicate samples, and are representative of three independent experiments. *P* < 0.0025 (rBT474) and *P* < 0.0008 (rSKBR3). **(B)** Western blot analysis showing steady-state pEGFR^**Y992**^, pHER3^**Y1197**^, pAkt^**Y308**^ phosphoprotein and cleaved PARP product expression in rBT474 and rSKBR3 cells after 72 hours of treatment with 5 μ*M* gefitinib, erlotinib, or neratinib (1 and 5 μ*M*), or vehicle (0.01% DMSO) alone. Steady-state actin protein levels served as a control for equal loading of protein. The results are representative of three independent experiments.

Gefitinib and erlotinib are FDA-approved EGFR TKIs [40-42]. In our hands, when used at a final concentration of 5 μ*M*, neither drug was able to block persistent EGFR tyrosine phosphorylation in lapatinib-resistant cells, maintained in 1 μ*M* lapatinib, nor did they restore lapatinib sensitivity (Figure 6A, B). Neratinib, in contrast to lapatinib, gefitinib, and erlotinib is an irreversible EGFR and HER2 TKI [43]. Consistent with previous reports [43], we found that neratinib was a potent inhibitor of parental HER2^+^ breast cancer cells (see Additional file 7). Neratinib, when used at higher concentrations than in parental cell cultures, inhibited persistent phosphorylation of EGFR, HER3, and Akt^**T308**^ in resistant cells, triggering cell apoptosis (increased cleaved PARP), and inhibition of cell growth and viability (*P* < 0.0008 in rSKBR3; *P* < 0.0025 in rBT474) (Figure 6A, B). These findings suggest that persistent EGFR signaling, rather than incomplete inhibition HER2, can play a role in maintaining the lapatinib-resistant phenotype.

### Autoinduction of heregulin in resistant cells drives the EGFR-HER3-PI3K signaling axis

**Figure 7:**
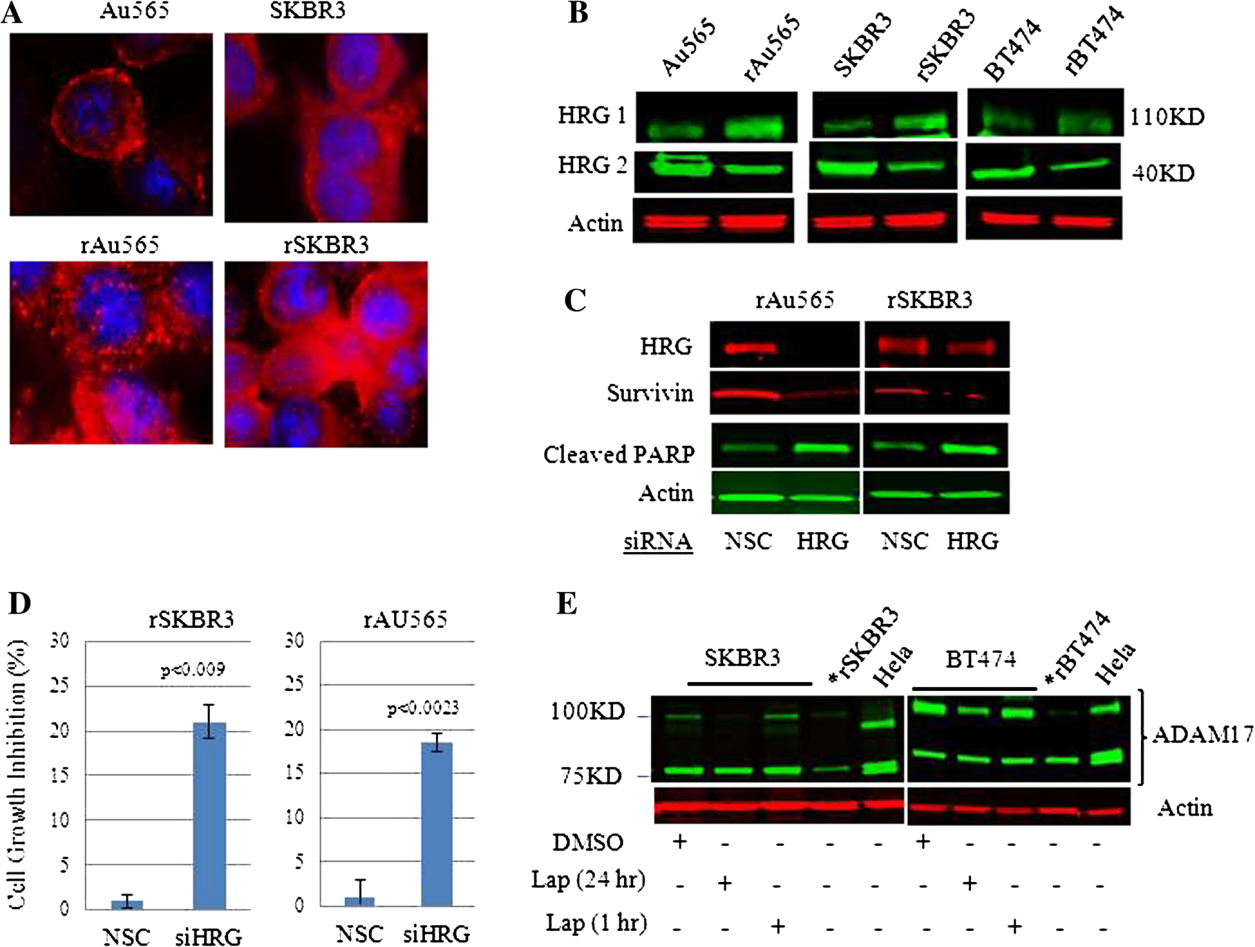
**Autocrine induction of HRG drives survival of resistant cells. (A)** Immunofluorescence microscopy of HRG expression in the indicated cell lines by using a primary rabbit anti-HRG antibody and visualized with an anti-rabbit IgG Alexa Fluor 555 (red) conjugated secondary antibody. Cell nuclei were visualized by DAPI staining (blue). These results are representative of other fields on the slide. **(B)** Western blot analysis of HRG type 1 (115 kDa) and type 2 (40 kDa) steady-state protein levels in lapatinib-resistant (rSKBR3; rAu565; rBT474) maintained in 1 μ*M* lapatinib, and untreated parental cell counterparts (SKBR3; BT474; Au565); actin served as a control for equal loading of protein. **(C)** Western blot analysis of survivin and cleaved PARP product after HRG knockdown in rAu565 and rSKBR3 cells. Cells transfected with scrambled siRNA construct (NSC) served as controls. Actin served as a control for equal loading of protein. **(D)** Effects of siRNA-mediated knockdown of HRG on tumor cell growth in rAu565 and rSKBR3 cell lines. Results represent the mean ± standard error of triplicate samples, and are representative of three independent experiments. *P* < 0.009 (rSKBR3) and *P* < 0.0023 (rAu565). **(E)** Western blot analysis of ADAM17 protein level in parental BT474 and SKBR3 ± lapatinib treatment as indicated in the figure. Resistant cells (rBT474 and rSKBR3) were growing in the presence of 1 μ*M* lapatinib. Hela cell extract was used as a positive control. Two bands from 75 kDa to 100 kDa can be detected by a specific ADAM17 antibody. These results are representative of three independent experiments.

We next sought to identify an underlying driver responsible for the persistent activation of the HER3-EGFR-PI3K signaling axis in lapatinib-resistant HER2^+^ breast cancer cells. Previous work from our laboratory had shown that heregulin β1 (HRG), a soluble ligand for HER3 and HER4, but not an EGFR ligand (EGF), can abrogate the inhibitory effects of lapatinib on cell-signaling pathways in parental HER2^+^ breast cancer cells [44,45], findings that were recently confirmed by Settleman and colleagues [46]. We therefore speculated that autocrine induction of HRG might play a role in the development of lapatinib resistance by providing the HER3 activation input, which, in conjunction with concomitant persistent EGFR activation, results in the formation of HER3-EGFR heterodimers. As shown, HRG protein expression was indeed increased in lapatinib-resistant cells compared with parental cell counterparts (Figure 7A, B). In contrast, we did not find increased expression of EGF ligands (data not shown). Interestingly, we found the 105-kDa membrane-bound species (Figure 6B, labeled HRG1), which can activate HER3 [47], to be the predominant form of HRG increased in resistant cells. Moreover, protein expression of the 40-kDa soluble form of HRG was decreased in resistant cells compared with parental cell counterparts (Figure 7B, labeled HRG2). Importantly, targeted molecular knockdown of HRG in resistant cells induced apoptosis (cleaved PARP) and decreased cell growth and viability (*P* < 0.009 in rSKBR3; *P* < 0.0023 in rAu565) (Figure 7C, D).

We next sought to gain a better understanding of the mechanism underlying the increased expression of membrane-bound HRG in resistant cells. Based on RT-PCR analysis, increased HRG resistant cells did not appear to be transcriptionally mediated (data not shown). ADAM17 is a metallopeptidase that proteolytically processes the 105-kDa membrane-bound form to smaller-molecular-weight soluble forms of HRG [48]. A previous report suggested that transient inhibition of Akt phosphorylation in trastuzumab-treated HER2^+^ breast cancer cells can lead to increased expression of ADAM17 and consequently increased expression of the lower-molecular-weight (40 kDa) soluble form of HRG [49]. In contrast, here we showed that the major forms of ADAM17 were inhibited over time in lapatinib-treated parental HER2^+^ breast cancer cell lines (Figure 7E, compare 1-hour and 24-hour treatments). Furthermore, ADAM17 was markedly reduced in lapatinib-resistant cells compared with their untreated parental cell counterparts. These findings made it tempting to speculate that inhibition of ADAM17 by lapatinib blocks proteolytic processing of the 105-kDa membrane-bound form of HRG, leading to its increased expression and concomitant decreased expression of lower-molecular-weight forms in resistant cells.

### Increased *HRG* expression predicts a poor outcome in HER2^+^ breast cancer patients

**Figure 8:**
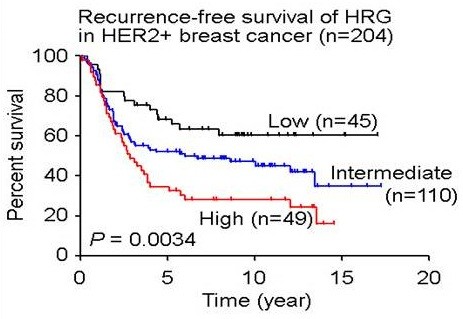
**Recurrence-free survival in HER2**^**+ **^**breast cancer patients according to HRG expression levels.** Tick marks in Kaplan-Meier estimates of recurrence-free survival indicate patients whose data were censored by the time of last follow-up or owing to death. *P* < 0.0034 was calculated by using log-rank Mantel-Cox test.

To shed light on the potential clinical implications of the autocrine induction of HRG in lapatinib-resistant HER2^+^ breast cancer cells, we analyzed the relation between HRG gene expression and clinical outcome in women with HER2^+^ breast cancer. Our analysis of the relation between *HRG* gene expression and clinical outcome in women with HER2^+^ breast cancer (*n* = 204) revealed a linear correlation between HRG expression and risk of recurrence (*P* = 0.0036, Cox-regression analysis) and a statistically significant difference (*P* = 0.0034, Kaplan-Meier Estimates survival analysis) between high HRG expression and decreased recurrence-free survival (RFS) (Figure 8). Median RFS in tumors with high expression and others (Intermediate plus Low) was 2.84 and 10.04 years, respectively. By using clinical parameters that were associated with clinical outcome, such as tumor size, grade, nodal status, HER2, ER, and PR status, we found that expressions of *HRG* was independently poor prognosis factor (comparing *HRG* high, intermediate, and low expression groups, *P* = 0.049, *n* = 581, COXPH survival analysis). Thus, autoinduction of HRG in lapatinib-resistant tumors could potentially contribute to a more-aggressive tumor phenotype with a poorer clinical outcome.

## Discussion

The robustness of a biologic system can be defined by its ability to maintain function when perturbed [50]. Accordingly, loss of HER2 signaling represents a significant perturbation to HER2-addicted breast cancer cells. Previous work from our laboratory and others has shown that the antitumor activity of lapatinib tracks with its ability to inhibit HER2 signaling [31,51]. Prolonged exposure to lapatinib, however, leads to the development of acquired therapeutic resistance in models of HER2^+^ breast cancer and in patients. We and others have shown that resistance to lapatinib does not appear to be mediated by reactivation of HER2 [17,23,32]. Instead, we now show that an autocrine feedback mechanism involving membrane-bound HRG can promote a previously unsuspected EGFR-HER3-PI3K-PDK1 signaling axis that is resistant to the effects of lapatinib and other FDA-approved EGFR TKIs. A key finding here is that the unopposed action of EGFR, which is incompletely inhibited by lapatinib, can transactivate HER3 in a manner driven by autocrine HRG. These findings demonstrate the robustness of the HER receptor-ligand system that enables HER2^+^ breast cancer cells to survive loss of HER2 signaling without the need to invoke mutations in the target kinase or its downstream intermediaries, or the activation of redundant signaling pathways.

In contrast to HRG, we were unable to demonstrate increased expression of EGFR ligands (for example, EGF betacellulin) in our models of lapatinib resistance. It is intriguing to speculate that the preferential induction of HRG reflects the drive of the tumor cell to maintain PI3K pathway activation in response to the loss of the HER2-HER3 oncogenic signaling complex, which is a potent PI3K pathway activator. Induction of EGFR ligands could have led to the formation of EGFR homodimers, which are less-potent activators of PI3K signaling compared with HER3-containing dimers. Indeed, lapatinib-resistant cells may also be primed to respond to HRG stimulation, as HER3, the cognate receptor for HRG, has been shown to be upregulated in HER2^+^ breast cancer models of acquired lapatinib resistance [21].

The mechanism involved in the autoinduction of HRG in resistant cells described here differs from the transient activation of HRG previously reported in trastuzumab-treated cells [49]. First, activation of HRG by trastuzumab was not shown to be directly linked to the development of acquired therapeutic resistance to trastuzumab. Second, induction of HRG in trastuzumab-treated cells was reportedly mediated by activation of ADAM17. In contrast, we showed that lapatinib inhibits expression of ADAM17, which may explain the increased expression of full-length membrane-bound HRG with a concomitant decrease in the expression of the lower-molecular-weight forms. Although lapatinib and trastuzumab both target HER2, our findings further underscore the distinct biologic effects that each can have on HER2^+^ targeted breast cancer cells.

The findings reported here highlight the importance of the cell context in the interpretation of predictive or correlative biologic readouts. For example, we previously reported that the phosphorylation state of HER3 could discriminate those patients with HER2^+^ inflammatory breast cancers who were more likely to respond to lapatinib monotherapy [16]. In that lapatinib-naïve setting, HER3 was likely transactivated by HER2 and therefore more sensitive to the antitumor activity of a potent HER2 tyrosine kinase inhibitor such as lapatinib. However, in HER2^+^ breast cancer cells that have become resistant to lapatinib, HER3 phosphorylation can be regulated by EGFR-HER3 dimers, which were not responsive to inhibition by lapatinib or other EGFR TKIs. Thus, monitoring tumors for the presence of increased HER3 phosphorylation, and perhaps phosphorylated EGFR, during lapatinib treatment may be an effective biomarker to identify patients whose tumors are becoming HRG-rewired. In addition, phosphorylation of Akt^**S473**^, which has long been considered a hallmark of PI3K pathway activation, was inhibited in lapatinib-resistant cells despite persistent activation of the PI3K pathway. An explanation for this apparent discrepancy can be attributed to the increased expression of a PI3K-mTOR regulated phosphatase (PHLPPL) that dephosphorylates Akt on S473, in lapatinib-resistant cells (Additional file 6A). Thus, the predictive power of biomarkers such as phosphorylated HER3 or phospho-Akt^**S473**^ would need to be placed into the context of the signals regulating its activation for clinical implementation. Consequently, clinical confirmation of the predictive nature of the elucidated pathway biomarker architecture would have to take place within that same context: in this instance tumor tissue from patients who relapsed after initially responding to lapatinib therapy and not from more easily obtained pretreatment biopsy samples. Our findings provide the scientific rationale to collect these tumor specimens so that validation of biomarkers of acquired resistance could be rigorously interrogated.

We previously showed that the antitumor activity of lapatinib in HER2^+^ breast cancer cells was not affected by EGF stimulation [44]. Here, however, increased expression of HRG can not only promote acquired therapeutic resistance to HER TKIs, but it also can mediate primary resistance to lapatinib (see Additional file 8) [44-46]. The frequent expression of HRG in solid tumors [52], including triple-negative breast cancers, may provide an explanation as to why current FDA-approved HER TKIs have had limited clinical impact in the treatment of the majority of HER2-overexpressing and EGFR-expressing solid tumors, with the exception of head and neck cancers [53]. Importantly, we identified HRG expression as an independent negative predictor of clinical outcome in patients with HER2^+^ breast cancers (Figure 8). Thus, targeting ligand-mediated feedback loops represents a new treatment strategy to overcome therapeutic resistance established through this mechanism.

Although current FDA-approved EGFR TKIs did not suppress HRG-driven EGFR activation in our models of resistance, siRNA-mediated knockdown of EGFR and treatment with the irreversible pan-HER TKI neratinib exerted antitumor effects in resistant cells (Figure 6A, B). Furthermore, whereas HRG can reverse the antitumor effects of lapatinib in parental HER2^+^ breast cancer cells (Additional file 8) [44-46], the antitumor effects of neratinib in parental HER2^+^ breast cancer cells are more resistant to HRG (Additional file 8). These findings are consistent with the ability of neratinib to exert antitumor effects on HRG-expressing resistant cells. Although neratinib is described as a pan-HER inhibitor, at clinically relevant concentrations, it can affect non-HER receptor kinases that contain homologous ATP kinase domains. Whereas lapatinib has been shown to be a highly specific TKI for HER2 and EGFR, neratinib and many other FDA-approved TKIs exhibit promiscuous inhibitory effects on non-HER kinases at clinically relevant concentrations [54]. These effects may contribute to the antitumor effects of neratinib in resistant cells, particularly at higher concentrations. Indeed, preliminary clinical data indicate that neratinib remains clinically active in the treatment of HER2^+^ breast cancers that have progressed on prior lapatinib-based therapy (Chow L, *et al*. Efficacy and safety of neratinib (HKI-272) in combination with paclitaxel in HER2^+^ metastatic breast cancer. San Antonio Breast Cancer Symposium, 2010). Furthermore, it is not surprising that parental HER2^+^ breast cancer cells were more sensitive to the antitumor effects of neratinib compared with lapatinib-resistant cells. Resistance to HER2 TKIs does not appear to be mediated by one underlying mechanism, as we and others have shown [17-23,55,56]. Therefore, completely reversing established resistance will likely require more than a single targeted intervention (for example, neratinib). It will require a combination approach, which, based on the findings reported here, should include inhibitors that block HRG-HER3-EGFR-PI3K-PDK1 signaling. These findings suggest that inhibition of wild-type EGFR remains an attractive therapeutic strategy awaiting the development of more-effective EGFR inhibitors.

The findings presented here have broad implications for the development of TKIs used to treat cancer and other kinase-driven diseases. As we have demonstrated, selection of clinical candidates based on activity profiles from *in vitro* kinase assays can be misleading. To the extent that lapatinib, erlotinib, and gefitinib are considered potent EGFR kinase inhibitors, none was able to neutralize HRG-mediated activation of EGFR. In contrast, neratinib appears to be a more-effective inhibitor of EGFR phosphorylation and activation, even in the presence of HRG in resistant and parental cells. It is tempting to suggest that the use of PI3K or mTOR selective inhibitors will prevent the development of ligand-mediated resistance. However, given the complex feedback mechanisms that govern these cytoplasmic signaling events, and the potential for HRG to exert promiscuous effects on cell-signaling pathways in a PI3K-independent manner [57], combination therapies that target both proximal and distal signaling are more likely to yield better clinical outcomes. Progressing TKIs into the clinic, based on their ability to inhibit multiple tyrosine autophosphorylation sites, may lead to the identification of more-effective drugs with a reduced risk of developing therapeutic resistance, and better candidates for personalized, combination therapies.

## Conclusions

Molecular targeted therapies that are directed against tyrosine kinases and receptor tyrosine kinases represent an important class of cancer drugs. However, development of TKI resistance remains a significant clinical dilemma that has limited the clinical impact of this class of targeted drugs in a broad range of solid tumors against which they were predicted to be effective. Past descriptions of mechanisms of TKI resistance have been attributed to mutations in targeted kinases or compensatory activation of signaling pathways that circumvent the target. Here we demonstrated the robustness of the HER biologic system to respond to a significant perturbation in cell signaling in the context of describing an entirely new mechanism of resistance to HER TKIs, including the FDA-approved dual HER2/EGFR TKI lapatinib, which is triggered by autocrine induction of the HER3 ligand, heregulin β1 (HRG). Whereas lapatinib, a supposed equipotent HER2 and EGFR kinase inhibitor, based on data from *in vitro* kinase assays, appropriately inhibited HER2 signaling, EGFR conversely was incompletely inactivated. Persistent EGFR signaling, coupled with the autocrine induction of membrane-bound HRG, contributed to a switch in the regulation of cell survival from HER2-HER3-PI3K in treatment-naïve HER2^+^ breast cancer cells to an HRG-driven EGFR-HER3-PI3K-PDK1 signaling axis in lapatinib-resistant tumor cells. Importantly, the FDA-approved EGFR TKIs gefitinib and erlotinib failed to block EGFR signaling and restore lapatinib sensitivity. Wild-type EGFR did, however, remain an attractive target, as molecular knockdown of EGFR and treatment with the irreversible pan-HER TKI neratinib blocked residual EGFR signaling, exerting an antitumor effect in resistant cells.

We further showed the clinical relevance of increased HRG expression in TKI-resistant tumor cells in a large breast cancer dataset (*n* = 204) of women with HER2^+^ breast cancers where increased HRG expression was an independent predictor for a significantly poorer clinical outcome (recurrence-free survival) compared with women whose tumors expressed moderate to low levels of HRG (*P* < 0.0036). Thus, incomplete inhibition and persistent signaling of the target itself, driven by a ligand-mediated autocrine feedback loop, may have broad implications for the treatment of diseases by using TKI therapies. These findings underscore potential inadequacies associated with the current approach of selecting clinical TKI candidates based on activity profiles from *in vitro* kinase assays. If incomplete target inhibition driven by autocrine ligand induction can mediate resistance to a selective inhibitor, such as lapatinib, then induction of ligand-driven autocrine feedback loops in response to promiscuous kinase inhibitors may be a new major causal factor of resistance. Selecting clinical lead candidates based on their ability to inhibit multiple tyrosine autophosphorylation sites instead of inhibition from *in vitro* kinase assays may lead to the identification of more-effective drugs with a reduced risk of developing therapeutic resistance.

## Abbreviations

4EBP1: eIF4E-binding protein 1; ADAM17: ADAM Metallopeptidase 17; Akt: Protein kinase B; ALLTO: Adjuvant lapatinib and/or trastuzumab treatment optimization; ATP: Adenosine triphosphate; EGF: Epidermal growth factor; EGFR: Epidermal growth factor receptor; FDA: Food and Drug Administration; HER2: Human epidermal growth factor receptor 2; HER2+: HER2 overexpression; HER3: Human epidermal growth factor receptor 3; HER4: Human epidermal growth factor receptor 4; HRG: Heregulin β1; mTOR: Mammalian target of rapamycin; p70S6K: p70S6 kinase; PARP: Poly (ADP-ribose) polymerase; PDK1: Phosphoinositide-dependent kinase 1; PHLPPL: PH domain leucine-rich repeat-containing phosphatase-like; PI3K: Phosphoinositide 3-kinase; PI3KCA: Catalytic subunit of PI3K; PKC: Protein kinase C; PTEN: Phosphatase and tensin homolog; RPMA: Reverse phase protein microarray; RTK: Receptor tyrosine kinase; SGK: Serum-glucocorticoid regulated kinase; siRNA: Small interfering RNA; TKI: Tyrosine kinase inhibitor.

## Competing interests

EP and JW are shareholders and consultants for Theranostics Health Inc., which has licenses on aspects of the technologies used in this study. EP is a cofounder of Theranostics Health, Inc., and a member of their advisory board. This work was supported by grants from the Komen Foundation Scholars Program (N.L.S), the Sisko Foundation (N.L.S), the Balderacchi Gift (N.L.S), and the George Mason University College of Science (E.F.P.).

## Authors’ contributions

WX, SZ, LL, and WG maintained all of the cell cultures, designed and performed treatment interventions with small-molecule inhibitors, and ran SDS-PAGE Western blots. WX, SZ, LL, and XY designed and performed siRNA experiments. XY and TO helped SZ perform immunofluorescence microscopy. SB helped with data analysis and provided critical review of the manuscript. KL and QC mined the breast cancer database QC had generated for the HRG genomic data and provided statistical input on analysis. EP, JW, and RG performed and analyzed the RPMA studies. NLS conceived of the ideas of the manuscript. NLS, WX, and EP oversaw the conduct of the experiments, data analysis, and wrote the manuscript. NLS and EP provided funding for the experiments performed. All authors read and approved the manuscript for publication.

## Supplementary Material

Additional file 1 List of antibodies used in the RPMA analysis. Click here for file

Additional file 2 **Lapatinib-resistant cells remain viable in concentrations of lapatinib lethal to parental cells.** Cell growth and viability in parental cells (BT474; SKBR3) treated with 1 μ*M* lapatinib for 72 hours, compared with resistant cells (rBT474; rSKBR3) continuously cultured in the presence of 1 μ*M* lapatinib. Parental cells treated with vehicle alone (0.01% DMSO) served as controls. Results represent the mean ± standard error of triplicate samples, and are representative of three independent experiments. Click here for file

Additional file 3 **Persistent PI3K signaling in lapatinib-resistant cells.** Reverse-phase protein microarray analysis of the indicated phosphorylated proteins by using the indicated phosphotyrosine-specific antibodies. Results represent the mean ± standard error of triplicate samples and are representative of three independent experiments. Click here for file

Additional file 4 **Inhibition of c-src does not reverse lapatinib resistance.** Treatment of rBT474 and rSKBR3 with a specific src inhibitor (AZD0530) had relatively little effect on cell growth. Top panels show the activation of Src in rBT474 and rSKBR3 compared with parental cells after treatment with 1 μ*M* lapatinib for 24 hours, as demonstrated by Western blotting, by using a phosphospecific antibody to src^Y416^. Actin steady-state protein served as a control for equal loading of protein. The middle panels demonstrate inhibition of src^Y416^ by a specific src kinase inhibitor AZD0530, used at 10 μ*M* for 72 hours, and the lower bar graph shows the effects of AZD0530 on the growth of rBT474 and rSKBR3 cells. Resistant cells were maintained in the presence of 1 μ*M* lapatinib. Results represent the mean ± standard error of triplicate samples, and are representative of three independent experiments. Click here for file

Additional file 5 **Inhibition of c-met does not reverse lapatinib resistance.** rBT474 and rSKBR3 cells were treated with a selective c-met inhibitor, SU11274 at 10 μ*M* for 72 hours and then analyzed for cell viability and proliferation. Resistant cells were maintained in the presence of 1 μ*M* lapatinib. Results represent the mean ± standard error of triplicate samples, and are representative of three independent experiments. Click here for file

Additional file 6 **EGFR knockdown and inhibition of PI3K/mTOR signaling block PHLPPL2 protein expression. (A)** The effects of EGFR knockdown on steady-state levels of the indicated proteins/phosphoproteins was determined by using specific primary antibodies (see Methods). The absolute optical density (OD) values attributed to the PHLPPL2 protein, determined by using the Odyssey Infrared Imaging System, are indicated under each corresponding lane. **(B)** Inhibition of PI3K/mTOR signaling blocks PHLPPL2 protein expression. The effects of 0.2 μ*M* NVP-BEZ 235 for 48 hours on the expression of PHLPPL2 protein in rBT474 and rSKBR3 cells, as determined by Western blot. Actin protein levels served as a control to ensure equal loading of protein. Resistant cells were maintained in the presence of 1 μ*M* lapatinib. Parental cells (BT474; SKBR3) were treated with 1 μ*M* lapatinib for 24 hours. Results are representative of three independent experiments. Click here for file

Additional file 7 **Sensitivity of parental HER2**^**+**^** breast cancer cells line to neratinib.** Parental BT474 and SKBR3 were treated with neratinib at the indicated concentrations (0.1; 0.5; 1; 5 μ*M*) for 72 hours, and then growth and viability were determined. Results represent the mean ± standard error of triplicate samples, and are representative of three independent experiments. Click here for file

Additional file 8 **HRG reverses the antitumor effects of lapatinib, but not neratinib, in parental HER2**^**+**^** breast cancer cells.** Cell growth and viability was assessed in parental BT474 cells treated with 1 μ*M* lapatinib or neratinib in the presence or absence of HRG (50 ng/ml) for 72 hours. Cells treated with vehicle alone (0.01% DMSO) served as controls. Results represent the mean ± standard error of triplicate samples, and are representative of three independent experiments. Click here for file
