# Supplementary figures and images for: An heregulin-EGFR-HER3 autocrine signaling axis can mediate acquired lapatinib resistance in HER2+ breast cancer models
Source: Breast Cancer Res. 2013 Sep 18;15(5):R85. doi: 10.1186/bcr3480 (PMC3978995; doi:10.1186/bcr3480)

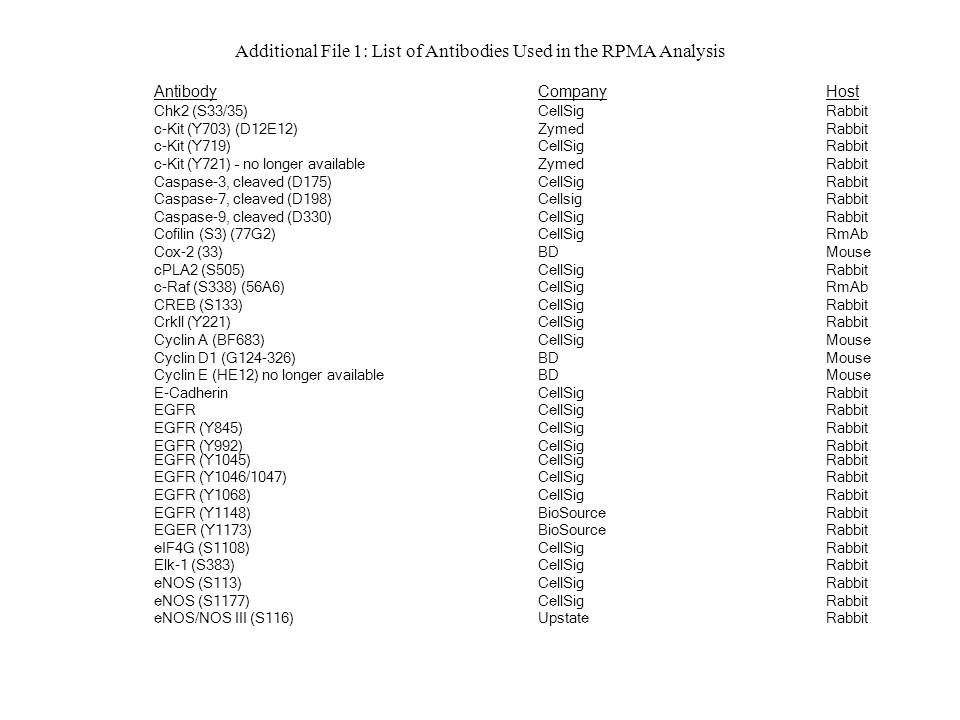

Supplement: Additional file 1 — List of antibodies used in the RPMA analysis. [file bcr3480-S1.zip › BCR Additional file 1 Oct 2013.jpg]

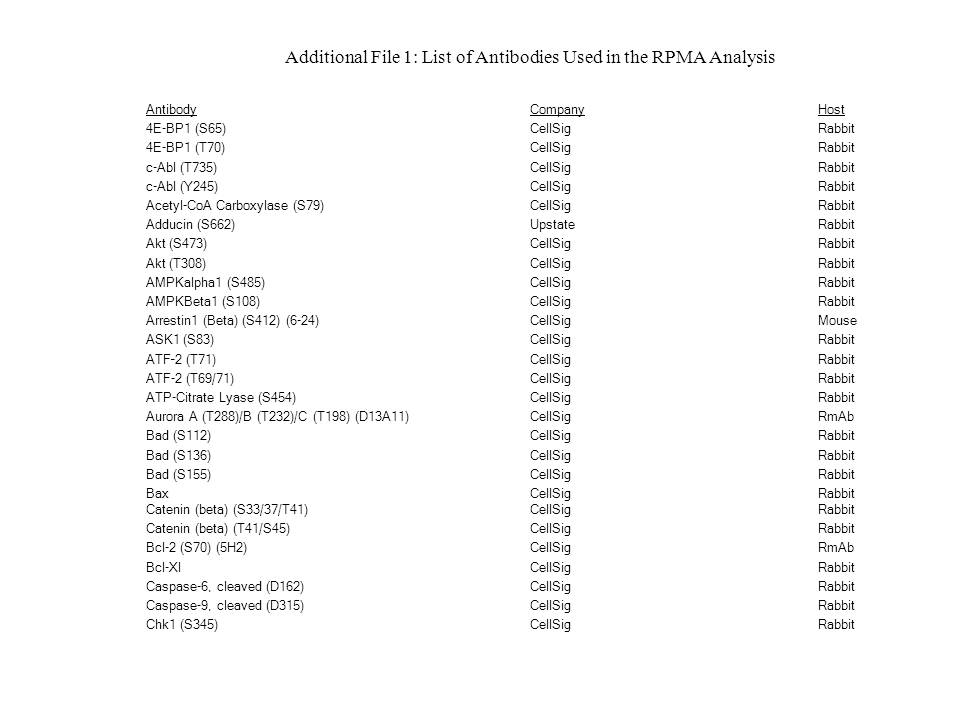

Supplement: Additional file 1 — List of antibodies used in the RPMA analysis. [file bcr3480-S1.zip › BCR Additional file 1b Oct 2013.jpg]

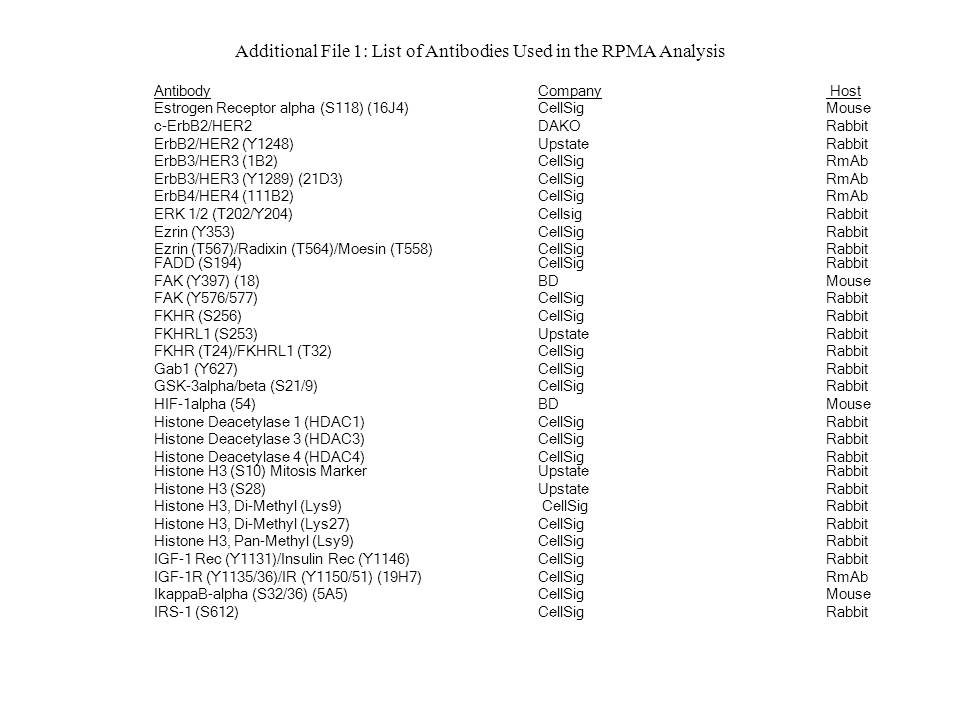

Supplement: Additional file 1 — List of antibodies used in the RPMA analysis. [file bcr3480-S1.zip › BCR Additional file 1c Oct 2013.jpg]

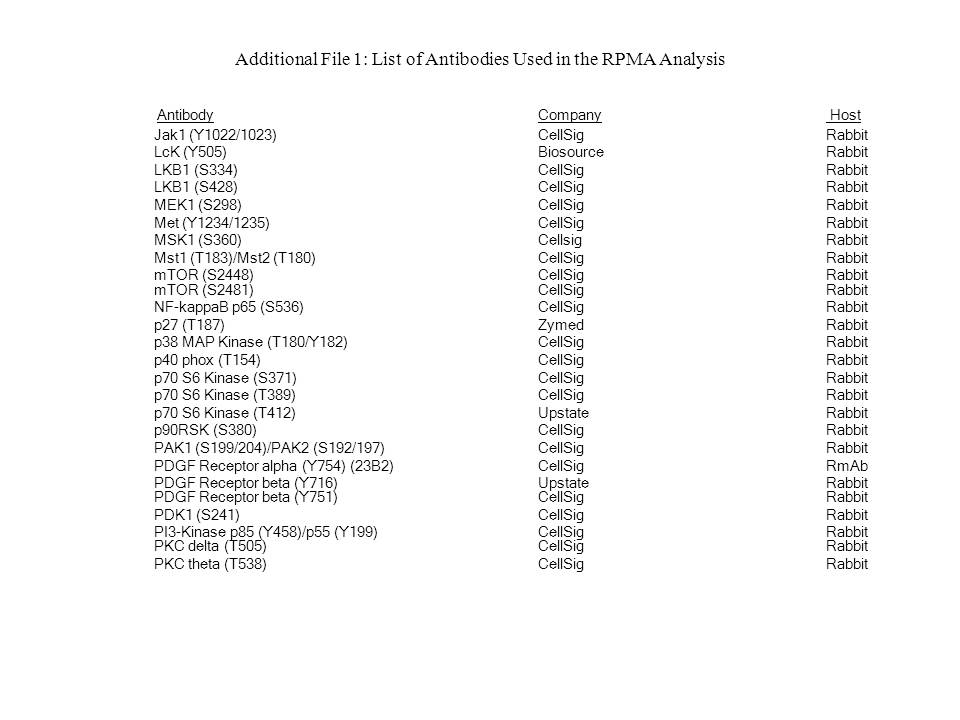

Supplement: Additional file 1 — List of antibodies used in the RPMA analysis. [file bcr3480-S1.zip › BCR Additional file 1d Oct 2013.jpg]

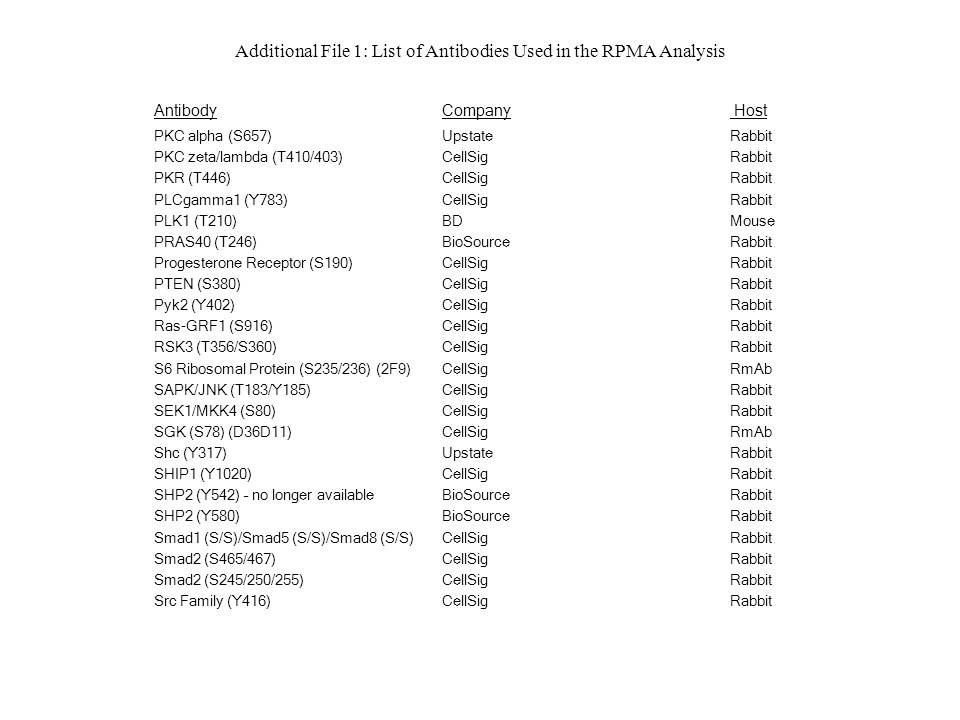

Supplement: Additional file 1 — List of antibodies used in the RPMA analysis. [file bcr3480-S1.zip › BCR Additional file 1e Oct 2013.jpg]

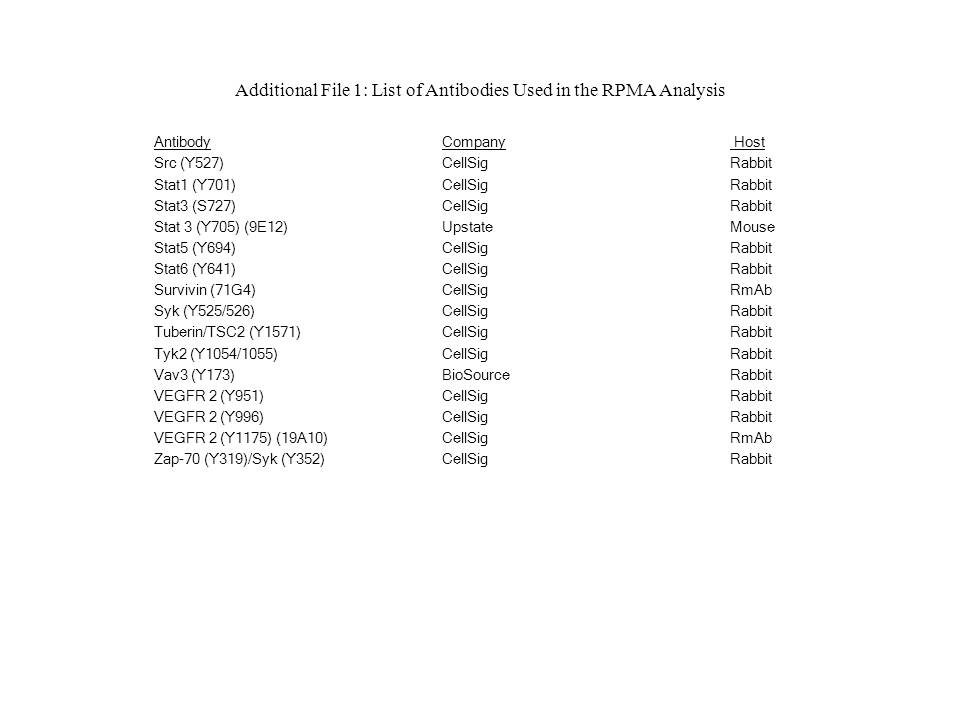

Supplement: Additional file 1 — List of antibodies used in the RPMA analysis. [file bcr3480-S1.zip › BCR Additional file 1f Oct 2013.jpg]

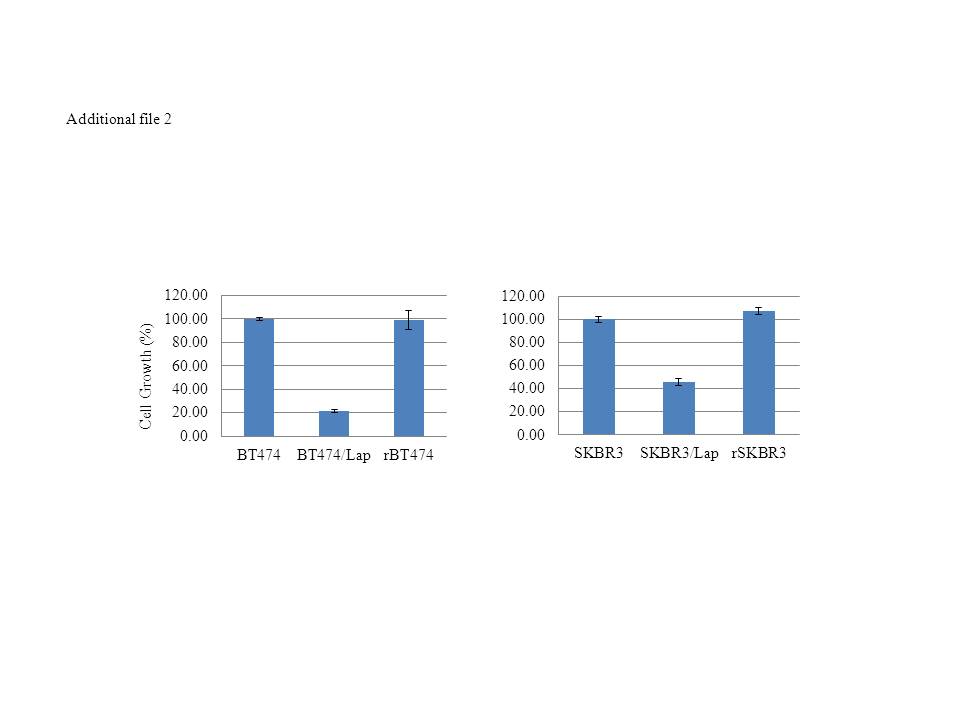

Supplement: Additional file 2 — Lapatinib-resistant cells remain viable in concentrations of lapatinib lethal to parental cells. Cell growth and viability in parental cells (BT474; SKBR3) treated with 1 μM lapatinib for 72 hours, compared with resistant cells (rBT474; rSKBR3) continuously cultured in the presence of 1 μM lapatinib. Parental cells treated with vehicle alone (0.01% DMSO) served as controls. Results represent the mean ± standard error of triplicate samples, and are representative of three independent experiments. [file bcr3480-S2.jpeg]

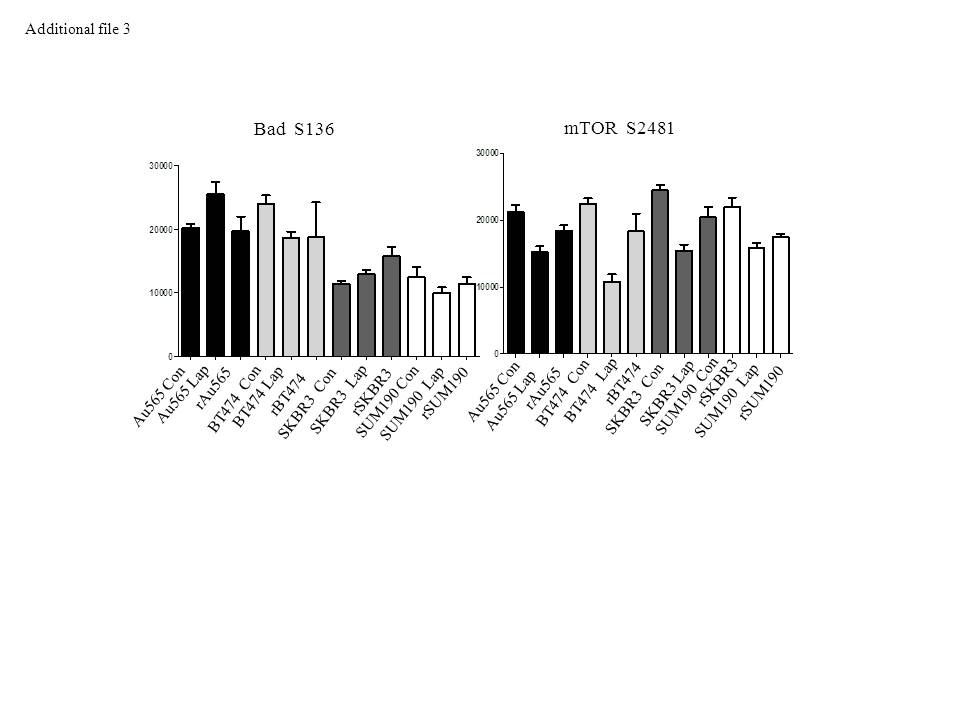

Supplement: Additional file 3 — Persistent PI3K signaling in lapatinib-resistant cells. Reverse-phase protein microarray analysis of the indicated phosphorylated proteins by using the indicated phosphotyrosine-specific antibodies. Results represent the mean ± standard error of triplicate samples and are representative of three independent experiments. [file bcr3480-S3.jpeg]

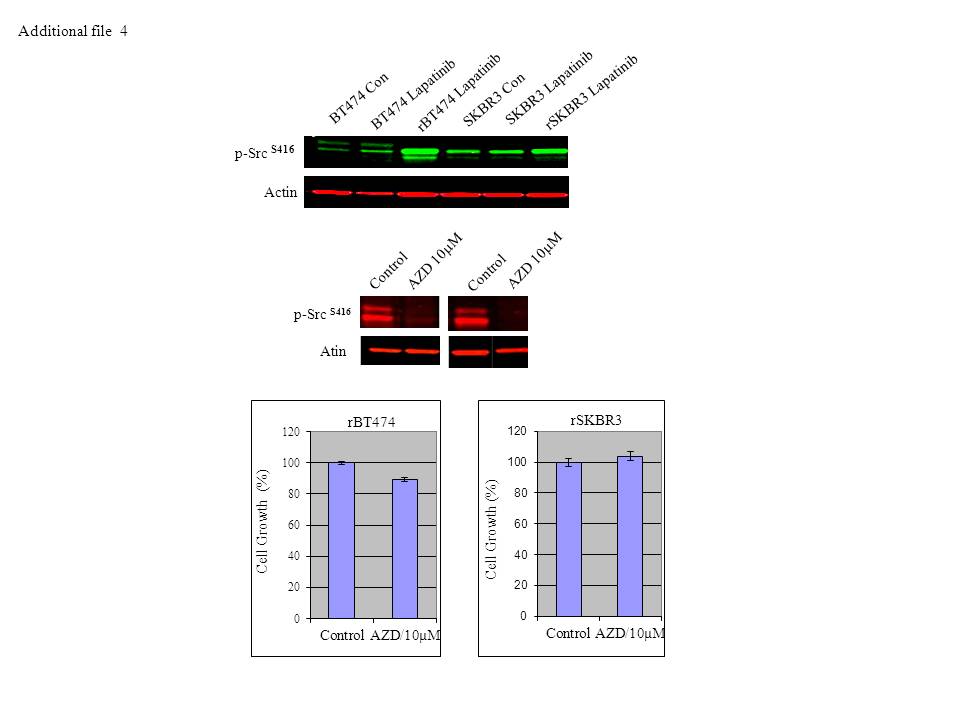

Supplement: Additional file 4 — Inhibition of c-src does not reverse lapatinib resistance. Treatment of rBT474 and rSKBR3 with a specific src inhibitor (AZD0530) had relatively little effect on cell growth. Top panels show the activation of Src in rBT474 and rSKBR3 compared with parental cells after treatment with 1 μM lapatinib for 24 hours, as demonstrated by Western blotting, by using a phosphospecific antibody to srcY416. Actin steady-state protein served as a control for equal loading of protein. The middle panels demonstrate inhibition of srcY416 by a specific src kinase inhibitor AZD0530, used at 10 μM for 72 hours, and the lower bar graph shows the effects of AZD0530 on the growth of rBT474 and rSKBR3 cells. Resistant cells were maintained in the presence of 1 μM lapatinib. Results represent the mean ± standard error of triplicate samples, and are representative of three independent experiments. [file bcr3480-S4.jpeg]

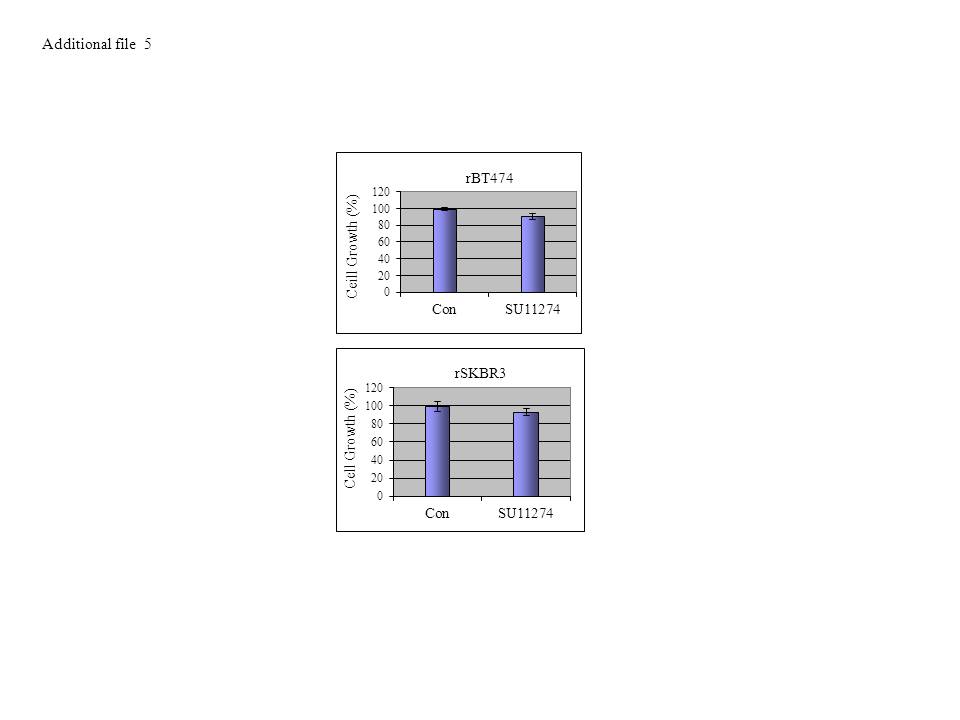

Supplement: Additional file 5 — Inhibition of c-met does not reverse lapatinib resistance. rBT474 and rSKBR3 cells were treated with a selective c-met inhibitor, SU11274 at 10 μM for 72 hours and then analyzed for cell viability and proliferation. Resistant cells were maintained in the presence of 1 μM lapatinib. Results represent the mean ± standard error of triplicate samples, and are representative of three independent experiments. [file bcr3480-S5.jpeg]

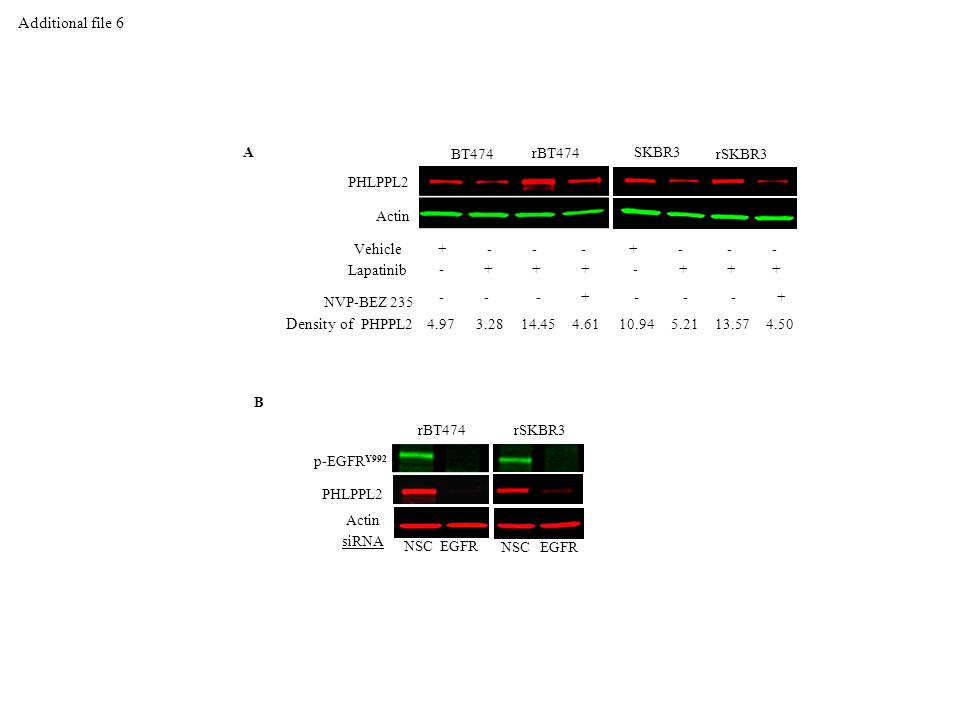

Supplement: Additional file 6 — EGFR knockdown and inhibition of PI3K/mTOR signaling block PHLPPL2 protein expression. (A) The effects of EGFR knockdown on steady-state levels of the indicated proteins/phosphoproteins was determined by using specific primary antibodies (see Methods). The absolute optical density (OD) values attributed to the PHLPPL2 protein, determined by using the Odyssey Infrared Imaging System, are indicated under each corresponding lane. (B) Inhibition of PI3K/mTOR signaling blocks PHLPPL2 protein expression. The effects of 0.2 μM NVP-BEZ 235 for 48 hours on the expression of PHLPPL2 protein in rBT474 and rSKBR3 cells, as determined by Western blot. Actin protein levels served as a control to ensure equal loading of protein. Resistant cells were maintained in the presence of 1 μM lapatinib. Parental cells (BT474; SKBR3) were treated with 1 μM lapatinib for 24 hours. Results are representative of three independent experiments. [file bcr3480-S6.jpeg]

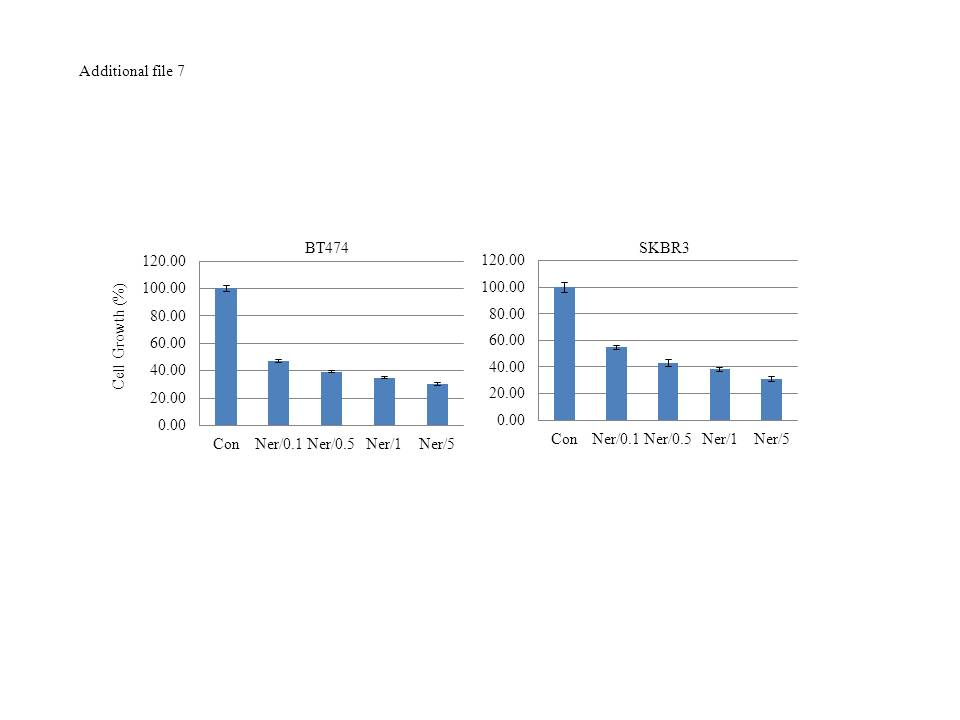

Supplement: Additional file 7 — Sensitivity of parental HER2+ breast cancer cells line to neratinib. Parental BT474 and SKBR3 were treated with neratinib at the indicated concentrations (0.1; 0.5; 1; 5 μM) for 72 hours, and then growth and viability were determined. Results represent the mean ± standard error of triplicate samples, and are representative of three independent experiments. [file bcr3480-S7.jpeg]

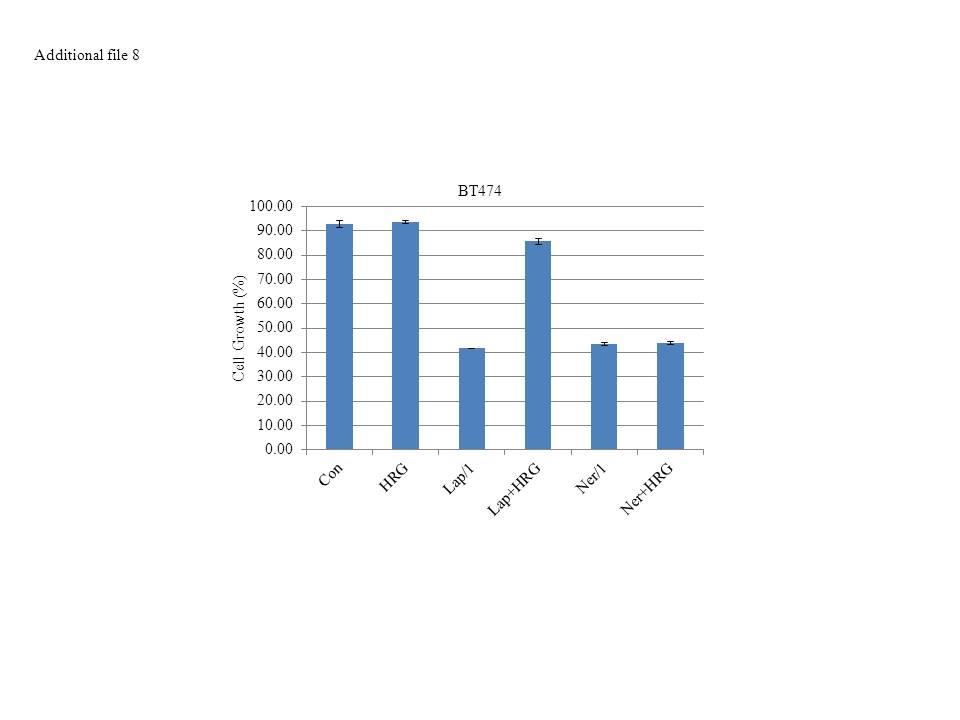

Supplement: Additional file 8 — HRG reverses the antitumor effects of lapatinib, but not neratinib, in parental HER2+ breast cancer cells. Cell growth and viability was assessed in parental BT474 cells treated with 1 μM lapatinib or neratinib in the presence or absence of HRG (50 ng/ml) for 72 hours. Cells treated with vehicle alone (0.01% DMSO) served as controls. Results represent the mean ± standard error of triplicate samples, and are representative of three independent experiments. [file bcr3480-S8.jpeg]
